# Continuous Molecular Monitoring Using Electrochemical Aptamer‐Based Sensors: Remaining Challenges for Long‐Term In Vivo Deployment

**DOI:** 10.1002/advs.77896

**Published:** 2026-09-27

**Authors:** Shibo Liu, Xiangling Li, Wei Ouyang

**Affiliations:** ^1^ Thayer School of Engineering Dartmouth College Hanover NH USA

**Keywords:** antifouling materials, bioresorbable materials, continuous molecular monitoring, electrochemical aptamer‐based biosensors, wireless electronics

## Abstract

In vivo continuous molecular monitoring represents a long‐standing, transformative objective in medicine, with the potential to fundamentally reshape disease diagnostics, therapeutic decision‐making, and long‐term patient management by providing dynamic biochemical information that is currently inaccessible. Electrochemical aptamer‐based biosensors offer a particularly promising and generalizable platform, as engineered aptamers can selectively bind diverse molecular targets and transduce binding‐induced conformational changes into robust electrochemical signals. Recent studies have demonstrated encouraging progress toward extended in vivo operation, including early examples of week‐scale functionality, highlighting the growing feasibility of this approach. Nevertheless, achieving robust and broadly applicable long‐term in vivo deployment remains an ongoing challenge due to factors such as sensor degradation and limitations in fully integrated device architectures. To address these challenges, multiple strategies have emerged, including drift‐canceling calibration, non‐natural nucleic acids, antifouling coatings, nanoengineered electrodes, advanced immobilization and ultralow‐power wireless systems, which are beginning to converge to enhance overall device performance. This review examines principles of aptamer‐based biosensing, delineates mechanisms of signal degradation, and surveys emerging strategies for stability enhancement and system‐level integration, outlining pathways toward longitudinal continuous molecular monitoring in personalized medicine.

## Graphical abstract

Continuous molecular monitoring using electrochemical aptamer‐based sensors enables real‐time tracking of dynamic biomarkers with transformative potential for precision medicine. Achieving clinically relevant month‐long stability remains a central challenge due to multiple degradation pathways. Integrated advances in calibration strategies, probe design, antifouling interfaces, nanoengineered electrodes, and system‐level electronics are converging to enable robust, fully integrated platforms for long‐term in vivo sensing.

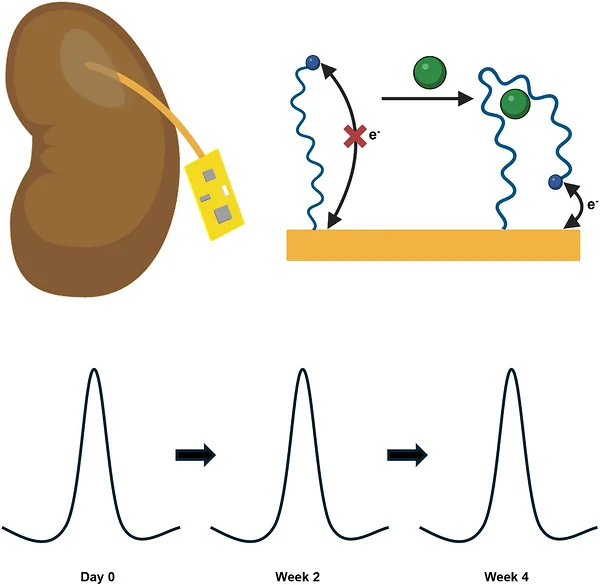

## 1 Introduction

Continuous, real‐time monitoring of molecular biomarkers inside the body, here referred to as continuous molecular monitoring (CMM), will fundamentally transform disease diagnostics, therapeutics and long‐term management (Figure 1a) [1, 2, 3]. Many physiologically and clinically relevant molecular signals are inherently dynamic, often fluctuating on timescales of minutes to hours, and therefore cannot be accurately captured by intermittent or discrete measurements [4, 5, 6]. For example, glucose levels exhibit rapid postprandial excursions and nocturnal fluctuations that are frequently missed by sparse sampling [7, 8], while drug concentrations can undergo sharp absorption and clearance phases that critically determine therapeutic efficacy and toxicity [9, 10]. Similarly, transient biochemical events, such as neurotransmitter release, can occur on sub‐millisecond timescales following action potential‐induced calcium influx and may therefore remain undetected without continuous monitoring [11, 12, 13]. As a result, intermittent measurements can obscure peak concentrations and misrepresent temporal trends, limiting the ability to resolve causal relationships between molecular dynamics and physiological outcomes. In this context, continuous monitoring provides a fundamentally different level of insight. For example, continuous assessment of cardiac troponin could enable earlier detection of myocardial infarction [14, 15, 16]. Similarly, real‐time tracking of chemotherapeutic drug levels, particularly within tumor microenvironments, could guide precision dosing in cancer therapy [17, 18]. For chronic conditions such as autoimmune diseases and inflammatory bowel disease, continuous monitoring of biomarkers like inflammatory cytokines could provide patients and clinicians with real‐time feedback on disease dynamics, improving day‐to‐day management and reducing reliance on episodic clinic visits [19, 20, 21].

**FIGURE 1:**
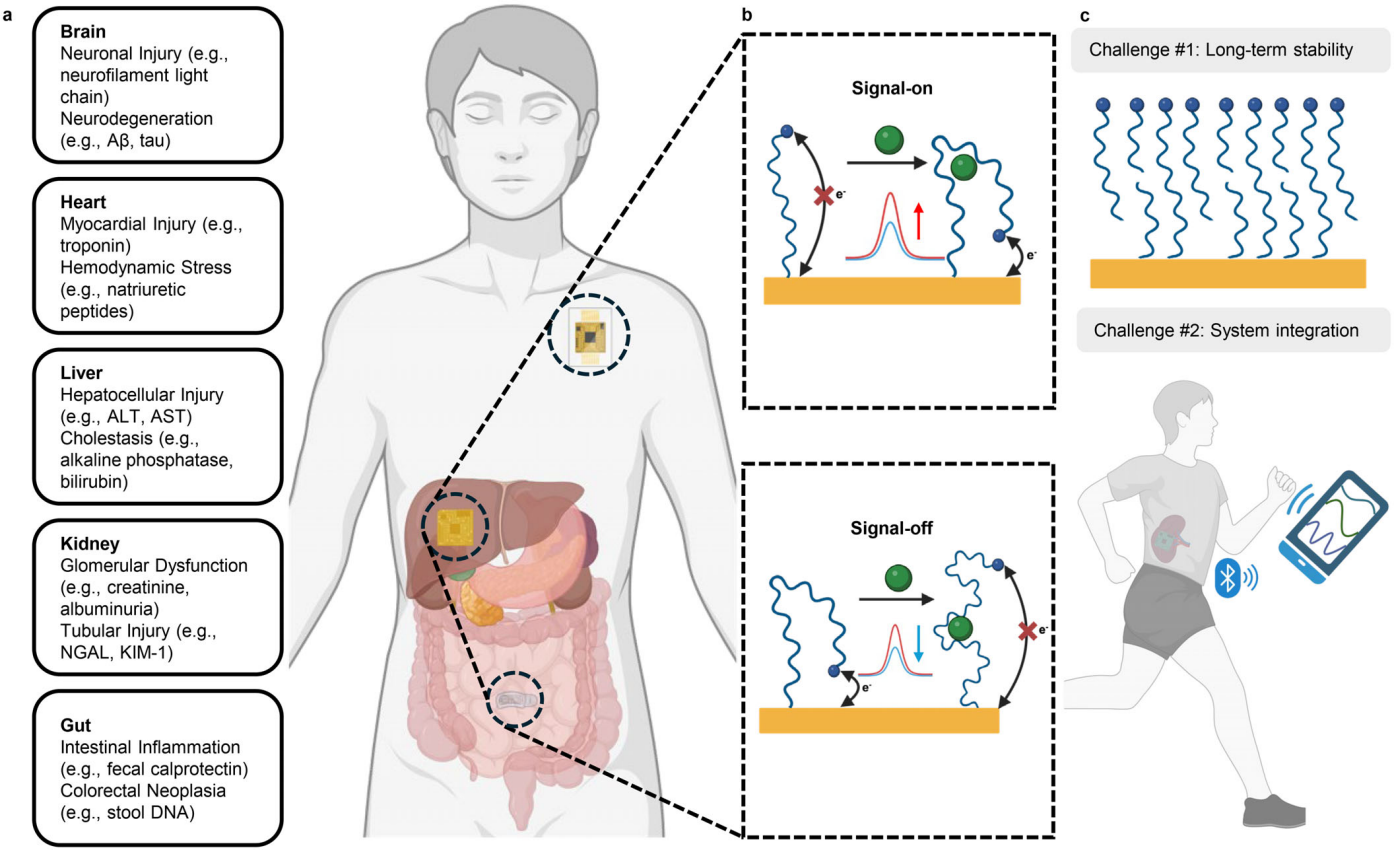
Overview of in vivo continuous molecular monitoring using aptamers. (a) Representative biomarkers across major organs. (b) Signal‐off and signal‐on mechanisms of electrochemical aptamer‐based sensors. Created in BioRender. Liu, S. (2026) https://BioRender.com/cxvtwgp. (c) Key challenges for longitudinal in vivo continuous molecular monitoring using aptamers. Created in BioRender. Liu, S. (2026) https://BioRender.com/cxvtwgp.

Achieving CMM hinges on several key capabilities. Sensors must maintain long‐term operational stability within complex physiological environments to ensure consistent signal fidelity over weeks or months [22, 23]. They must support repeated molecular recognition to enable continuous quantification [24, 25]. Furthermore, seamless integration with wireless electronics, power management modules and biocompatible encapsulation in a miniature, compliant form factor is essential for long‐term use without imposing undue burden on patients [26, 27]. The most advanced continuous glucose monitoring (CGM) patches, such as Dexcom G7 and Abbott FreeStyle Libre 3, serve as model systems that embody these desired capabilities. Their enzyme‐based electrochemical sensors, stabilized by antifouling coatings, provide ∼15 days of calibration‐free operation, while enzyme‐catalyzed reactions generate continuous electrochemical signals that follow real‐time changes in target concentration [28, 29, 30]. Full integration into a miniature, skin‐conformal patch further enables CGM in daily life, substantially improving the quality of life of patients with diabetes.

Despite this remarkable success, CGM remains limited to glucose detection [31, 32]. Achieving general CMM across a broader spectrum of molecular biomarkers, including small‐molecule drugs, metabolites, cytokines and hormones, remains an unrealized ideal [24, 33]. Many of these analytes lack enzyme‐mediated recognition pathways, necessitating alternative biorecognition chemistries that can operate stably and reversibly in complex physiological environments [34, 35, 36]. Among various emerging candidates, electrochemical aptamer‐based (EAB) sensors represent a particularly promising framework because their programmable nucleic‐acid architectures enable highly selective and reversible binding across diverse analytes, while target‐induced conformational switching yields a direct, reagent‐free electrochemical readout ideally suited for in vivo operation (Figure 1b) [37, 38]. Nevertheless, substantial challenges must be addressed before EAB sensors can support chronic in vivo monitoring (Figure 1c) [39, 40]. A major challenge arises from the fact that aptamers undergo multiple, intertwined degradation pathways such as probe desorption, nuclease hydrolysis, competitive displacement and protein adsorption that limit functional lifetimes to hours or days [41, 42, 43, 44].

Recent research in EAB sensors has begun to converge on integrated solutions spanning chemistry, materials and electronics [45, 46, 47, 48]. Calibration‐based strategies, such as kinetic differential measurement (KDM), provide compensates for baseline drift at the signal‐processing level [45, 46, 49]. Nuclease‐resistant nucleic acid analogues, such as xenonucleic acids (XNAs), further enhance probe stability by reducing enzymatic degradation and preserving aptamer functionality [47, 48]. Antifouling hydrogels and zwitterionic coatings form robust hydration barriers that suppress nonspecific adsorption and immune activation [50, 51, 52]. Nanostructured electrodes such as nanoporous gold and laser‐induced graphene improve surface area, enhance electron transfer and physically shield aptamers from fouling [17, 46, 53, 54, 55]. Covalent immobilization strategies including electrografting and thiol‐Michael addition create thiol‐free linkages that resist desorption [56, 57, 58, 59]. Ultralow‐power front‐end circuits, wireless communication and emerging bioresorbable platforms illustrate the feasibility of self‐contained in vivo EAB devices capable of chronic operation and safe resorption after use [60, 61, 62, 63, 64, 65, 66, 67, 68].

This review integrates advances across these domains to provide a comprehensive picture of progress toward stable, and fully integrated in vivo EAB sensors. By synthesizing drift‐canceling approaches, nuclease‐resistant nucleic acids, antifouling coatings, nanoengineered electrodes, covalent immobilization strategies, and integrated ultra‐low power electronics, we establish a cohesive framework for designing EAB sensors that can operate continuously in vivo. Ultimately, these developments chart a path toward long‐term CMM systems that could fundamentally reshape precision medicine.

## 2 Fundamentals of EAB Sensors

### 2.1 Sensing Principle

EAB sensors convert target recognition into an electrical signal through target‐induced conformational changes of redox‐labeled aptamers tethered to an electrode surface. The earliest realization of this concept, reported by Fan et al., established that hybridization‐ or binding‐induced structural rearrangements modulate the distance and dynamics between a redox tag and the electrode, thereby altering electron‐transfer efficiency [69]. Because the aptamer is immobilized and interrogated electrochemically, the EAB platform operates reagentlessly while maintaining remarkable specificity even in complex matrices. Depending on whether the conformational change moves the redox reporter away from or toward the electrode upon target binding, the resulting current modulation can manifest as either a signal‐off or signal‐on response.

#### 2.1.1 Signal‐Off Mechanism

The signal‐off mechanism relies on a decrease in electron‐transfer rate upon target binding. In these sensors, the probe adopts a flexible conformation that allows the redox tag to efficiently collide with the electrode in the absence of target. Hybridization or analyte binding rigidifies the structure by forming a duplex, G‐quadruplex, or tertiary fold, thereby increasing the tunneling distance and suppressing faradaic current [70]. This mechanism, often termed folding‐based or collision‐based signaling, was systematically analyzed by Xiao et al., who demonstrated that electron‐transfer modulation originates from changes in probe flexibility rather than simple mass adsorption [71]. Such folding‐induced current decreases typically yield signal suppressions of 60%–90%, depending on probe density and ionic conditions [71].

Despite their simplicity and robustness, signal‐off architectures exhibit intrinsic drawbacks. Because current suppression is bounded between 0% and 100%, the dynamic range and sensitivity are fundamentally limited [72]. Moreover, surface degradation, redox‐tag loss, or nonspecific fouling all reduce current, producing false‐positive signals indistinguishable from authentic binding events [72]. Under long‐term or in vivo operation, these one‐directional signal degradations accumulate and complicate calibration. Consequently, although signal‐off EAB sensors have been widely adopted as model systems for mechanistic study, their practical deployment in in vivo continuous monitoring remains challenging.

#### 2.1.2 Signal‐On Mechanism

To overcome the limitations of signal‐off mechanism, Xiao et al. introduced a signal‐on EAB design in which target binding brings the redox reporter closer to the electrode surface, producing a positive‐going current change [72]. In this configuration, a methylene blue (MB)‐labeled aptamer adopts an extended conformation in the absence of the target, keeping the reporter away from the electrode. Upon target binding, the aptamer folds into a compact structure such as G‐quadruplex that increases electron‐transfer collisions between the redox tag and the electrode. This binding event produces a several‐fold increase in current, corresponding to 270% signal gain for thrombin at 260 nM, an order of magnitude improvement over the 40% suppression observed in the preceding signal‐off architecture [72]. The extended dynamic range and positive‐going signal remarkably improve sensitivity and discrimination against surface degradation, since nonspecific fouling now leads to signal loss rather than apparent activation.

Subsequent theoretical and experimental studies further elucidated the kinetic origin of signal‐on responses. White et al. demonstrated that the direction of signal change depends not only on conformational rearrangement but also on the relative electron‐transfer rates of the bound and unbound states [73]. By tuning the frequency of square wave voltammetry (SWV), one can selectively enhance the contribution of the faster or slower state, thereby switching between signal‐off and signal‐on behavior for the same probe. This finding indicates that both architectures arise from a common mechanism in which target binding modulates probe flexibility and collision efficiency, rather than representing two fundamentally distinct phenomena.

Although signal‐on mechanism mitigates false‐positive drift and broadens detection range, they are not universally advantageous. The requirement for a cooperative conformational release restricts aptamer choices and the additional hybridization or strand‐displacement equilibria can compromise binding affinity and reusability [70, 72]. Thus, while signal‐on mechanism represents a major advance toward high‐gain, drift‐resistant EAB sensing, their performance ultimately depends on fine control of aptamer folding thermodynamics, hybridization kinetics and electrochemical interrogation conditions.

### 2.2 EAB‐Detectable Molecules

EAB sensors have demonstrated broad molecular recognition capabilities spanning small‐molecule drugs, metabolites and protein biomarkers. Their programmable selectivity and reversible binding make them uniquely suited for dynamic in vivo environments, where continuous and quantitative molecular tracking is required. Over the past decade, the portfolio of EAB‐detectable analytes has expanded from chemotherapeutics and antibiotics to metabolites and cytokines, highlighting their versatility across pharmacological and physiological domains. Table 1 summarizes representative examples of EAB sensors reported to date, detailing the demonstrated detection ranges, limits of detection (LOD), sample types and detection method for each analyte category. These studies collectively underscore the adaptability of the EAB architecture for monitoring chemically and structurally diverse molecular targets under in vivo conditions.

**TABLE 1:** Representative molecular targets for electrochemical aptamer‐based biosensing and their performance metrics.

Ref	Target Class	Target	Demonstrated Detection Range	Limit of Detection (LOD)	Sample type	Detection Method
[17]	Drugs	Doxorubicin	0.5–30 µM	—	In vitro (1xSSC buffer)	SWV
		Doxorubicin	3–10 µM	—	In vitro (fetal bovine serum)	
		Doxorubicin	∼0.7–2.4 µM	—	Ex vivo (tumor tissue)	
		Doxorubicin	∼0.6–2.1 µM	—	In vivo (mouse tumor)	
[49]	Drugs	Doxorubicin	0.01 µM–10 000 µM	10 nM	In vitro (1× SSC buffer)	SWV
		Doxorubicin	0.3–4 µM	—.	In vitro (human whole blood)	
		Doxorubicin	0.13–2.5 µM	—	In vivo (live rats)	
		Kanamycin	0.45–1.6 mM	—		
[76]	Drugs	Kanamycin	∼34–400 µM	—	In vivo (live rats)	SWV
		Doxorubicin	∼0.1–0.6 µM	—		
		Tobramycin	∼20–60 µM	—		
		Gentamicin	∼10–200 µM	—		
[77]	Drugs	Cocaine	1 µM–10 mM	—	In vitro (artificial CSF)	SWV
			∼30 µM–4 mM	—	In vivo (rat dorsal striatum)	
[78]	Drugs	Cocaine	∼0–1 µM	—	In vivo (rat jugular vein)	SWV
[87]	Metabolites	Phenylalanine	1 nM–3 mM	—	In vitro (cell culture media)	SWV
[88]	Metabolites	Glucose	0–50 mM	2.4 mM (upward)	Ex vivo (artificial ISF)	SWV
				7.44 mM (downward)		
		Lactate	0–20 mM	1.04 mM (upward)		
				4.43 mM (downward)		
[93]	Proteins	TNF‐α	0–2 ng/mL	—	In vitro (serum)	SWV
		IL‐6	0–30 ng/mL	—		
		IL‐8	0–30 ng/mL	—		
		TGF‐β1	0–150 pg/mL	—		
[94]	Proteins	IL‐6	N.R.	—	In vitro (DPBS)	SWV

#### 2.2.1 Drugs

Monitoring therapeutic drugs and drugs of abuse is of critical importance in both clinical pharmacology and public health. For therapeutics such as antibiotics and chemotherapeutics, maintaining drug concentrations within a narrow therapeutic window is essential to balance efficacy with toxicity, yet conventional sampling and laboratory assays provide only sparse snapshots of pharmacokinetics [10, 74, 75]. In vivo EAB sensors uniquely address this limitation by enabling continuous, real‐time molecular tracking directly in vivo. Early pioneering work by Ferguson et al. demonstrated a microfluidic electrochemical detector (MEDIC) integrating aptamer‐modified electrodes, continuous‐flow diffusion filtering and kinetic differential measurement for real‐time in vivo drug monitoring. In whole human blood, it accurately tracked therapeutic Doxorubicin (DOX) concentrations between 0.3 and 4 µM for over 4 h with deviations below 0.06 µM. When applied in vivo in rats, the sensor quantified DOX concentrations from 0.13 to 2.5 µM, showing excellent agreement with conventional assays. The same platform, when reconfigured with a kanamycin aptamer, detected concentrations of 0.45–1.6 mM in rats. Both sensors provided sub‐minute temporal resolution and maintained drift below 2% through KDM, underscoring the robustness and modularity of the MEDIC design [49]. Subsequently, Arroyo‐Currás et al. extended this approach to awake, freely moving rats by employing polysulfone‐encased EAB sensors for direct, real‐time monitoring of drugs in whole blood. These sensors enabled second‐scale pharmacokinetic measurements, establishing the feasibility of high‐temporal‐resolution drug monitoring in freely moving subjects [76]. More recently, Seo et al. developed flexible nanoporous gold electrode arrays capable of intratumoral monitoring of DOX [17]. In SSC buffer, the sensors exhibited a linear detection range of 0.5–30 µM, while in undiluted fetal bovine serum, they showed reproducible responses between 3 and 10 µM and stable operation for over 16 h. When implanted into B16‐F10 melanoma tumors in live mice, the sensors detected ∼0.6–2.1 µM DOX in real time with second‐scale temporal resolution and remained stable for more than 2 h. These in vivo measurements revealed striking disparities between plasma and tumor pharmacokinetics that would be invisible to circulation‐based sampling [17].

Similarly, in vivo EAB sensors have been adapted to monitor illicit drugs such as cocaine. Early work demonstrated that aptamer‐functionalized neural recording probes could be implanted into the rat striatum to achieve sub‐5 s temporal resolution of cocaine dynamics and a broad detection range spanning ∼30 µM‐4 mM. The sensor maintained stable operation in vivo for nearly 3 h, enabling continuous measurement of both locally infused and systemically administered cocaine dynamics in brain tissue [77]. More recently, the development of high‐affinity DNA aptamers with nanomolar binding has enabled intravenous EAB sensors to resolve circulating cocaine concentrations with seconds‐level resolution [78]. Their ability to provide high temporal resolution pharmacokinetic profiles holds promise for precision dosing, personalized medicine and long‐term therapeutic management.

#### 2.2.2 Metabolites

Metabolites such as glucose, lactate and phenylalanine are central indicators of physiological and pathological states. Glucose monitoring is indispensable for diabetes management [32, 79, 80], lactate serves as a vital metabolic biomarker linking energy expenditure with brain‐body interactions [81, 82] and phenylalanine levels are clinically relevant in disorders such as phenylketonuria and in exercise‐related metabolic dysfunction [83, 84]. Conventional enzyme‐based sensors have dominated this space but are constrained by stability issues, the requirement for labile cofactors (e.g., NAD^+^, FAD) [85, 86] and limited applicability beyond a few well‐studied metabolites. In contrast, aptamers provide chemically robust and highly modular recognition elements that can be engineered against diverse targets, including metabolites lacking suitable enzymes. Their reversible binding enables reagentless, real‐time monitoring and supports continuous electrochemical sensing, thereby extending the scope of metabolic monitoring toward personalized healthcare and in vivo applications.

Recent advances highlight aptamer‐based platforms as versatile alternatives for metabolite monitoring. In cell culture systems, Yang et al. demonstrated that aptamer sensors can be miniaturized and multiplexed to continuously monitor pH and phenylalanine with high temporal resolution [87]. The phenylalanine sensor responded over the physiologically relevant range of 1 nM–3 mM, with accurate detection in the plasma concentration window of 50–100 µM and less than 10% variation. Importantly, the KDM method minimized drift and enabled reliable long‐term monitoring. The ability to track amino acid fluctuations directly in media addressed a long‐standing challenge in reproducing metabolic environments for cancer biology and drug discovery.

Focusing on in vivo applications, Bakhshandeh et al. developed the Wearable Aptalyzer, which integrates hydrogel microneedle arrays with redox‐reporter aptamers for minimally invasive detection of glucose and lactate in interstitial fluid [88]. The glucose sensor achieved a detection range of 0–50 mM with a LOD of 2.4 mM during upward calibration, while the lactate sensor operated across 0–20 mM with a LOD of 1.04 mM during upward calibration, both showing excellent selectivity against interferents such as ascorbate and uric acid. In vivo tests in healthy mice and diabetic rats demonstrated stable performance over multiple hours, with sensor outputs strongly correlating to gold‐standard blood assays.

Together, these studies emphasize that aptamer‐based electrochemical sensors can deliver both high‐resolution amino acid monitoring in controlled in vitro environments and minimally invasive, multi‐day metabolic tracking in vivo. This dual progress underscores their potential not only to expand the analyte space beyond enzyme‐accessible metabolites but also to provide the operational stability and versatility required for future in vivo biosensors.

#### 2.2.3 Proteins

Protein biomarkers such as cytokines play central roles in inflammation, infection and disease progression [89, 90, 91]. Current clinical assays, including ELISA and chemiluminescent immunoassays, provide high sensitivity but remain labor‐intensive and are incompatible with continuous in vivo use [92]. In vivo EAB sensors offer a pathway toward real‐time protein monitoring, with the potential to capture dynamic fluctuations of protein biomarkers directly in living systems. Compared to antibody‐based probes, aptamers are more stable, chemically accessible and readily interfaced with miniaturized electronics, positioning them as attractive candidates for in vivo biosensors.

Recent demonstrations have underscored both the promise and the limitations of this approach. Gao et al. reported a flexible wound care platform integrating EAB sensors for cytokines including Tumor Necrosis Factor‐Alpha (TNF‐α), Interleukin‐6 (IL‐6), Interleukin‐8 (IL‐8) and Transforming Growth Factor‐Beta 1 (TGF‐β1) [93]. The platform enabled multiplexed, reagentless detection in murine wound models and human patients, achieving less than 5% drift over four weeks in buffer and faithfully tracking cytokine dynamics in vivo across several days. In a separate study, Huang et al. developed an IL‐6 aptamer sensor for monitoring inflammation in the colon [94]. The device exhibited reversible signal changes (40% in buffer) but faced rapid signal degradation in mucosal environments, losing up to 60% within 24 h and 30%–50% within 5 h in implanted rat models. Encapsulation with PVA‐MA hydrogel significantly improved stability, reducing signal degradation by 93% and extending operational lifetimes.

These studies highlight the feasibility of aptamer‐protein sensing but also reveal critical challenges. Unlike small molecules, proteins diffuse more slowly due to their larger size and lower diffusion coefficients, leading to reduced collision frequency with surface‐tethered aptamers and slower establishment of binding equilibrium [23, 95]. As a result, the conformational switching and electron‐transfer events upon target binding often yield weaker electrochemical signals. Achieving high affinity while retaining reversibility is particularly difficult. The very low k_off_ values required for pM‐nM K_D_ hinder real‐time responsiveness, limiting continuous readout [53]. In vivo, these kinetic constraints are confounded by the low abundance of target proteins and the presence of abundant serum proteins and metabolites, which exacerbate nonspecific adsorption and signal drift. Overcoming these obstacles will require a combination of aptamer engineering to tune binding kinetics, antifouling surface chemistries to preserve signal fidelity and circuit‐level amplification strategies to enhance sensitivity [96]. With these innovations, in vivo EAB sensors may ultimately transform protein biomarker monitoring from single‐point assays into continuous, clinically actionable readouts.

## 3 Challenges and Advances Towards Achieving Long‐Term Stability

### 3.1 Mechanism of Signal Degradation

Long‐term stability represents a fundamental requirement for reliable continuous monitoring over extended periods, which, however, has been a critical challenge for in vivo EAB sensors. Existing EAB sensors typically function for only a few hours in vivo, limiting their clinical utility as implantable devices. The mechanism of signal degradation involves several distinct factors, including electrochemical interrogation, passive desorption, competitive displacement of self‐assembled monolayers (SAMs), biofouling and enzymatic degradation. These mechanisms rarely occur in isolation but instead act synergistically to degrade sensor performance. A clear understanding of these mechanisms is therefore essential for developing strategies that extend EAB sensors lifetime and achieving long‐term molecular monitoring in vivo.

#### 3.1.1 Electrochemical Interrogation

Electrochemical interrogation itself is an important driver of signal degradation in EAB sensors. Repeated voltametric cycling places the monolayer under both electrical and chemical stress and two distinct modes of degradation emerge depending on whether the sensor is biased negatively or positively relative to the reference electrode (Figure 2a).

**FIGURE 2:**
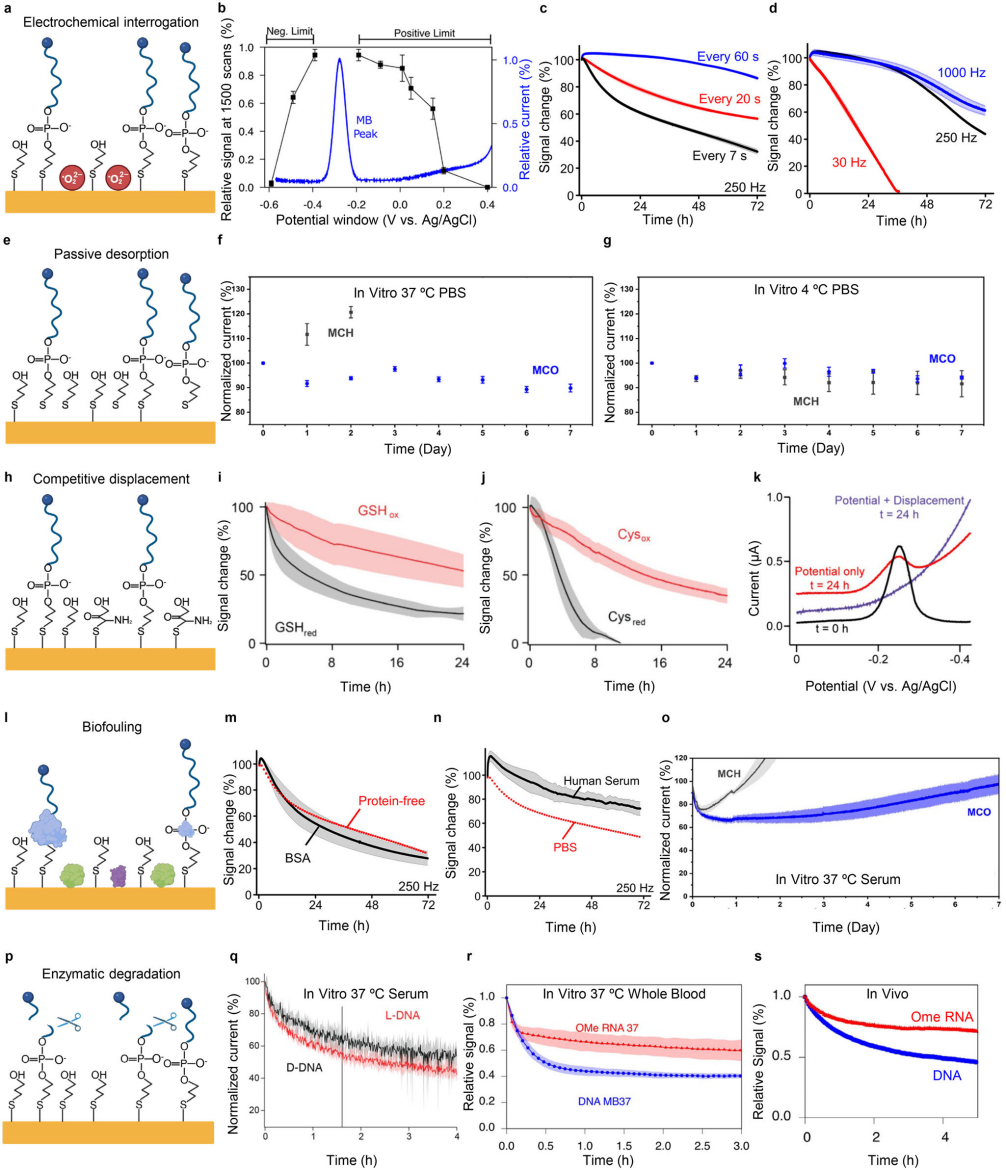
Mechanisms of signal degradation in EAB sensors. (a–d) Electrochemical interrogation. (a) Repetitive voltammetric interrogation induces thiol‐gold bond cleavage and the generation of reactive oxygen species (ROS). Created in BioRender. Liu, S. (2026) https://BioRender.com/trcx8cy. (b) Degradation accelerates outside a narrow potential window, with rapid signal loss occurring below −0.2 V where oxygen reduction generates ROS and above +0.2 V where gold oxidation begins. The methylene blue (MB) redox potential sits close to this safe region but does not fall entirely within it. Adapted with permission [41]. Copyright 2021, American Chemical Society. (c) Lowering interrogation frequency reduces cumulative damage; interrogations every 60 s produce substantially less degradation than those applied every 20 s or every 7 s. Adapted with permission [42]. Copyright 2023, American Chemical Society. (d) Higher square‐wave voltammetry (SWV) frequencies (1000 Hz versus 250 Hz or 30 Hz) improve signal stability by minimizing residence time at electrochemically damaging potentials. Adapted with permission [42]. Copyright 2023, American Chemical Society. (e‐g) Passive desorption. (e) Thermal desorption promotes ligand detachment from defect‐rich domains of self‐assembled monolayers (SAMs), even without interrogation. Created in BioRender. Liu, S. (2026) https://BioRender.com/trcx8cy. (f) Shorter‐chain backfilling molecules (MCH) rapidly degrade at 37°C in serum, whereas longer‐chain backfilling molecules (MCO) provide improved packing and slower degradation. Adapted with permission [44]. Copyright 2023, American Chemical Society. (g,) Desorption is substantially suppressed at 4°C, underscoring the temperature dependence of SAMs stability. Adapted with permission [44]. Copyright 2023, American Chemical Society. (h–k) Competitive displacement. (h) Thiolated molecules in biofluids nucleophilically exchange with thiol‐gold bonds. Created in BioRender. Liu, S. (2026) https://BioRender.com/trcx8cy. (i) Continuous voltammetric interrogation in 10 µM glutathione shows faster signal degradation in the reduced state than in the oxidized state. Adapted with permission [42]. Copyright 2023, American Chemical Society. (j) Exposure to 500 µM cysteine causes markedly faster signal degradation in the reduced form compared with the oxidized form. Adapted with permission [42]. Copyright 2023, American Chemical Society. (k) In voltammetric measurements, competitive displacement produces substantial loss of MB peak amplitude and elevated background current, consistent with SAMs removal and increased gold exposure. Adapted with permission [44]. Copyright 2023, American Chemical Society. (l–o) Biofouling. (l) Adsorption of serum proteins restricts aptamer motion and disrupts electron transfer. Created in BioRender. Liu, S. (2026) https://BioRender.com/trcx8cy. (m) Large proteins such as BSA accelerate short‐term signal loss, whereas in protein‐free buffer, degradation proceeds more slowly. Adapted with permission [42]. Copyright 2023, American Chemical Society. (n) In whole serum, rapid protein corona formation partially shields the interface, temporarily delaying degradation relative to PBS. Adapted with permission [42]. Copyright 2023, American Chemical Society. (o) MCO mitigates fouling and prolongs stability in serum over 7 days. Adapted with permission [44]. Copyright 2023, American Chemical Society. (p–s) Enzymatic degradation. (p) Schematic of nuclease‐mediated cleavage of surface‐bound aptamers, leading to signal loss. Created in BioRender. Liu, S. (2026) https://BioRender.com/trcx8cy. (q) Signal decay of L‐DNA (red) and D‐DNA (black) in serum in vitro. Similar behavior indicates nucleases are not the dominant cause of signal loss. Adapted with permission [99]. Copyright 2021, American Chemical Society. (r) Signal decay of nuclease‐resistant OMe RNA (red) and DNA (blue) in whole blood in vitro. Significant signal loss persists despite nuclease resistance, suggesting fouling dominates early degradation. Adapted with permission [41]. Copyright 2021, American Chemical Society. (s) In vivo stability in rats. OMe RNA (red) shows improved signal retention compared to DNA (blue), indicating a greater role of nuclease degradation in vivo. Adapted with permission [48]. Copyright 2024, Wiley‐VCH GmbH.

Leung et al. demonstrated that confining the voltage window to −0.4 to −0.2 V resulted in only about 5% signal loss after 1500 scans [41]. This range was selected to accommodate the redox peak of MB, which appears at −0.24 to −0.26 V in neutral phosphate buffer, while remaining narrow enough to minimize both reductive and oxidative desorption of the SAMs (Figure 2b). Subsequent work by Clark et al. further refined this understanding by showing that potentials more negative than −0.2 V drive oxygen reduction, leading to local generation of peroxide species, while potentials more positive than +0.2 V promote gold oxidation, both of which destabilize the SAMs [42]. This conclusion was later confirmed by direct mechanistic evidence. Using scanning electrochemical microscopy (SECM), Clark et al. demonstrated that interrogation at −0.2 V already generates hydrogen peroxide near the electrode surface at concentrations of around 30 µM and wider excursions down to −0.4 V increase the local concentration up to 320 µM, which are sufficient to damage the SAMs [43].

Although this stricter stability window of −0.2 to +0.2 V minimizes degradation, it excludes the MB redox transition and is therefore not practical for real sensing. To address this trade‐off, Clark et al. systematically evaluated how electrochemical interrogation parameters impact sensor stability under continuous monitoring [42]. By varying interrogation frequency and SWV parameters such as waveform frequency and voltage window, they demonstrated that the time sensors spend at damaging potentials dictates the rate of signal degradation. For example, when sensors are interrogated every 7 s by SWV at 250 Hz with an amplitude of 50 mV across the window from −0.1 to −0.5 V versus the reference electrode, they lost more than twice as much current over 72 h as those interrogated once per minute under otherwise identical conditions, highlighting that more frequent sensor biasing at damaging potentials accelerates signal degradation (Figure 2c). Similarly, waveform frequency strongly influences degradation. High‐frequency waveform at 1000 Hz preserved more than 60% of the initial current after 72 h, while low‐frequency waveform at 30 Hz produced rapid signal degradation, leading to complete signal loss at about 30 h (Figure 2d). The difference arises because slower waveforms prolong the residence time of the electrode at damaging negative potentials, thereby amplifying reductive stress on the SAMs [42, 97]. These results established interrogation waveform design not only just electrochemical probing but also as a key engineering lever for extending EAB sensors lifetimes.

Watkins et al. added a contrasting perspective by showing that electrochemical interrogation can also stabilize monolayers under certain conditions [44]. In week‐long experiments at 37°C, sensors that were scanned frequently maintained more stable blocking layers than those interrogated only once per day. This effect arises because repeated reductive interrogation suppresses thiol oxidation into disulfides, which otherwise drives thermal desorption of monolayer elements. Thus, while frequent scanning at damaging potentials accelerates probe loss, appropriately chosen interrogation protocols can paradoxically extend monolayer lifetimes by maintaining thiols in their reduced form.

Taken together, these studies show that interrogation‐induced degradation is not simply a function of scan count but is strongly determined by interrogation frequency, voltage window, and waveform frequency. Minimizing time at damaging potentials and avoiding excursions outside the −0.2 to +0.2 V dramatically improves stability, but this window does not accommodate the redox reporter peak such as that of MB, making it impractical for sensing applications. At the same time, the protective role of scanning in suppressing thiol oxidation highlights that interrogation can be both a source of degradation and a tool for stabilization. These insights suggest that extending the operational lifespan of EAB sensors will require carefully designed interrogation schemes matched to the electrochemical environment.

#### 3.1.2 Passive Desorption

Passive desorption constitutes a key degradation pathway in EAB sensors, stemming from the intrinsic instability of alkylthiolate SAMs under physiological conditions. Even in the absence of continuous electrochemical interrogation, Au‐S bonds and the lateral packing interactions within the SAMs are vulnerable to thermally driven and solute‐mediated destabilization (Figure 2e). Watkins et al. dissected these effects in multiday studies at 37°C using a once‐per‐day interrogation protocol, thereby minimizing electrochemical stress and isolating thermal contributions [44]. Their results revealed that desorption is strongly dictated by structural defects and intermolecular forces. Molecules positioned at grain boundaries or step edges experience weakened van der Waals stabilization, lowering the activation barrier for desorption. These defect‐rich regions thus act as initiation sites for SAMs loss, a process exacerbated at body temperature where thermal energy accelerates bond rupture and molecular release.

The data highlighted a pronounced temperature dependence. At 37°C in bovine serum, 6‐mercapto‐1‐hexanol (MCH)‐based sensors degraded within 3 days, losing discernible redox‐tag peaks and exhibiting pronounced increases in background current, indicative of extensive passivation‐layer desorption and exposure of bare gold to parasitic reactions such as oxygen reduction (Figure 2f). By contrast, under identical interrogation at 4°C, robust voltammograms were preserved for at least 7 days, confirming that reduced thermal energy markedly slows desorption kinetics (Figure 2g).

Molecular design further modulates this degradation. Sensors employing longer‐chain alkylthiolates such as 8‐mercapto‐1‐octanol (MCO) displayed improved stability relative to conventional MCH due to stronger van der Waals interactions between adjacent molecules that increase the activation barrier for desorption. Nevertheless, even MCO layers failed within 3 days at 37°C, underscoring the severity of thermally driven desorption in physiological conditions. Collectively, these findings establish passive desorption as a substantial degradation pathway for EAB sensors operating at body temperature and highlight the importance of defect minimization and monolayer engineering as partial mitigation strategies.

#### 3.1.3 Competitive Displacement of SAMs

In addition to thermal desorption, EAB sensors are also compromised by competitive displacement of SAMs, a chemically driven process in which endogenous thiols in biofluids exchange with gold‐bound monolayer elements (Figure 2h). Unlike passive desorption, which is thermally driven, competitive displacement is a chemically mediated process whereby small‐molecule thiols in solution, such as cysteine and glutathione, nucleophilically attack Au‐S bonds and displace alkylthiol‐modified oligonucleotides or passivating molecules from the electrode surface.

Experimental evidence clearly shows how strongly these molecules influence sensor stability. Clark et al. demonstrated that when sensors were incubated in biofluid mimetics containing reduced cysteine or glutathione at elevated concentrations, signal loss was dramatic and rapid with complete disappearance of voltammetric peaks within a day (Figure 2i,j) [43]. By contrast, their oxidized counterparts produced slower degradation, consistent with the lower nucleophilicity of disulfides (Figure 2i,j). These data highlight that the redox state of physiological thiols critically influences displacement rates, with reduced forms being the most aggressive. The effect of thiol exchange is further emphasized when considered alongside electrochemical cycling [43]. In buffered solutions alone, sensors show a moderate decline in current under repeated interrogation. Yet when free thiols are present, the degradation is substantially accelerated, confirming that electrochemical stress and biochemical exchange act in concert to destabilize the sensing layer. Representative voltammograms reveal this synergy. In the presence of thiols, redox‐tag currents collapse far more quickly than in their absence, while background capacitive currents rise, indicative of monolayer desorption (Figure 2k).

Taken together, these findings suggest that competitive displacement may contribute to EAB sensor degradation in thiol‐containing biological media. However, current evidence is derived primarily from in vitro studies, and its relevance to in vivo degradation remains to be established.

#### 3.1.4 Biofouling

Biofouling is another major contributor to EAB sensors degradation. When they are placed in protein‐rich media, nonspecific adsorption of albumin and other macromolecules alters electron‐transfer kinetics and restricts aptamer flexibility (Figure 2l). Clark et al. further revealed that the rate of signal degradation due to biofouling depends strongly on the surrounding medium [42]. In protein‐free buffered saline, degradation is driven primarily by voltage‐induced desorption and proceeds at a moderate pace, with sensors retaining 40% of their initial current after 72 h (Figure 2m). However, the presence of bovine serum albumin (BSA) accelerates signal loss to below 30% over the same period, consistent with restricted probe conformational freedom and destabilization of the monolayer through nonspecific adsorption (Figure 2m). In contrast, sensors in undiluted human serum preserved 70% of their initial current after 72 h, significantly higher than in Phosphate Buffered Saline (PBS) (40%), as the rapid formation of a protein corona partially shielded the interface from voltage‐induced damage, even though competitive displacement by endogenous thiols still contributed to signal loss (Figure 2n).

Watkins et al. extended these observations by examining sensors in undiluted bovine serum for up to one week at 37°C [44]. They showed that proteins initially act as a passivating layer, slowing background current growth within the first several hours, but later promote accelerated monolayer desorption. For example, sensors with conventional MCH passivation rapidly lost discernible redox‐tag currents within 3 days, whereas those with longer‐chain MCO preserved 60% of their initial signal even after 7 days (Figure 2o). This difference arises because adsorbed foulants provide an energetic bridge that lowers the barrier for alkylthiolate release, thereby hastening probe loss for MCH, while the stronger intermolecular interactions in MCO layers render them more resistant. Consequently, serum can appear to stabilize sensors in the first hours, while ultimately exacerbating degradation during multiday operation.

These results demonstrate that biofouling has a dual character. Short‐term adsorption may mask electrode defects and suppress parasitic reactions, whereas long‐term protein rearrangements compromise both the structural integrity of the monolayer and the mobility of surface‐bound aptamers.

#### 3.1.5 Enzymatic Degradation

Enzymatic cleavage of aptamers by nucleases has long been considered a potential source of signal degradation for EAB sensors [42, 44, 98]. Since nucleic acids are susceptible to hydrolysis in biological fluids, nucleases may progressively digest surface‐bound aptamers, leading to the loss of redox‐reporter‐modified strands and a consequent reduction in measurable signal (Figure 2p).

However, whether nuclease activity dominates signal degradation is strongly context‐dependent. Shaver et al. evaluated nuclease‐resistant mirror‐image L‐DNA and natural D‐DNA aptamers in diverse biological fluids in vitro [99]. In serum, despite the inherent resistance of L‐DNA to nuclease hydrolysis, both constructs exhibited nearly identical biphasic signal degradation, with a rapid initial degradation followed by stabilization at approximately 40% of the original signal over 4 h (Figure 2q). These results indicate that, under such in vitro conditions, nuclease activity is not the primary driver of signal loss. Instead, interfacial processes, including SAM desorption and surface fouling, dominate signal degradation.

Consistent with this interpretation, Leung et al. further probed the contribution of enzymatic degradation in whole blood at 37 degrees [41]. Chemical regeneration of the sensor surface using urea recovers up to 80% of the signal after the initial degradation, indicating that this early‐stage signal loss is largely reversible and therefore dominated by surface fouling rather than irreversible enzymatic cleavage. To further assess the role of nucleases, the authors employed an enzyme‐resistant 2’O‐methyl RNA (OMe RNA). Despite its strong resistance to nuclease degradation, this construct still exhibits a pronounced exponential degradation phase (Figure 2r). This observation, consistent with prior reports on spiegelmers, further supports the conclusion that nuclease activity is not the primary driver of the early‐stage signal loss in vitro.

Importantly, these conclusions are derived from in vitro experiments, where the physicochemical environment may differ substantially from that encountered in vivo. Recent in vivo studies using XNAs have reported markedly improved stability. Leung et al. fabricated EAB sensors with OMe RNA exhibit substantially reduced signal loss compared to their DNA counterparts when deployed in the jugular vein of live rats [48]. Specifically, over 4 h of in vivo residence, OMe RNA‐based sensors retain approximately 70% of their initial signal, whereas DNA‐based sensors degrade to about 50% under the same conditions (Figure 2s). This suggests that, in vivo, enzymatic degradation of aptamers becomes more apparent once interfacial degradation is no longer overwhelmingly dominant.

Taken together, these findings indicate that nuclease activity is neither negligible nor solely responsible for signal loss. Rather, its apparent contribution depends strongly on experimental context, particularly the relative rates of fouling, monolayer degradation, and enzymatic cleavage. Distinguishing between these regimes is therefore essential when interpreting stability data and translating in vitro results to in vivo performance.

### 3.2 Strategies for Achieving Long‐Term Stability

#### 3.2.1 Calibration‐Based Strategies

Calibration‐based strategies, including KDM, dual‐reporter, and dual‐aptamer approaches, improve the long‐term reliability of EAB sensors by correcting signal drift rather than preventing its underlying physical causes. These methods do not directly mitigate degradation processes such as biofouling, monolayer instability, or nuclease‐mediated cleavage. Instead, they rely on internal referencing or drift‐insensitive features extracted from the electrochemical response to enable more stable and quantitative readout over time. Among these, KDM represents one of the most widely adopted and well‐established strategies for drift cancellation.

##### 3.2.1.1 Kinetic Differential Measurement Strategy for Drift Cancellation

KDM, first introduced by Ferguson et al., is a calibration‐based strategy that mitigates signal drift in EAB sensors by exploiting the frequency‐dependent electron transfer behavior of redox‐labeled aptamers [49]. In this approach, the sensor is interrogated at two SWV frequencies. As illustrated in Figure 3a, at high frequencies, the target‐bound state produces higher current (signal‐on), whereas at lower frequencies it yields lower current relative to the unbound state (signal‐off). This behavior arises from differences in electron transfer kinetics coupled with depletion effects at longer interrogation timescales. Crucially, while the target‐dependent signals differ between frequencies, background drift arising from processes such as fouling or monolayer degradation produces similar responses at both frequencies. KDM exploits this by computing a normalized differential signal, typically defined as the difference between the signal‐on and signal‐off responses divided by their average, thereby suppressing shared drift while preserving the target‐dependent component. In practice, two frequencies are selected that produce distinct, often opposite, target responses while exhibiting matched baseline drift.

**FIGURE 3:**
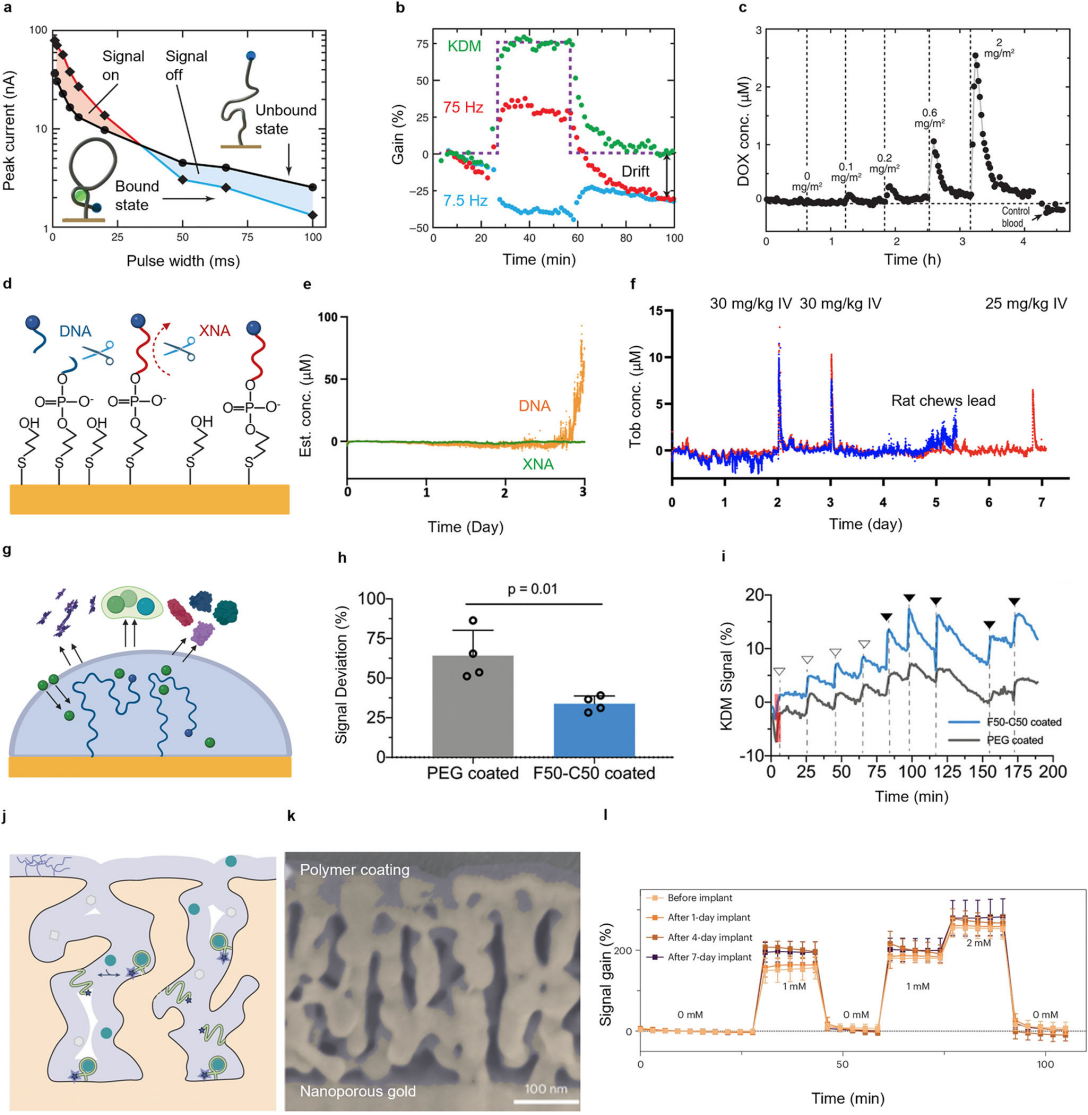
Strategies to enhance long‐term stability of EAB sensors. (a–c) Kinetic differential measurement (KDM) for drift correction. (a) Frequency‐dependent signal‐on/off behavior of redox‐labeled aptamers. Adapted with permission [49]. Copyright 2013, American Association for the Advancement of Science. (b) Drift suppression by combining signals at two frequencies. Adapted with permission [49]. Copyright 2013, American Association for the Advancement of Science. (c) In vivo drug monitoring enabled by KDM with minimal drift. Adapted with permission [49]. Copyright 2013, American Association for the Advancement of Science. (d–f) Non‐natural nucleic acids for enhanced probe stability. (d) Comparison of DNA and XNA aptamers. Created in BioRender. Liu, S. (2026) https://BioRender.com/v0nqlge. (e) Improved signal stability of XNA sensors over time. Adapted with permission [47]. Copyright 2026, American Chemical Society. (f) Long‐term in vivo monitoring of drug concentrations using XNA sensors. Adapted with permission [47]. Copyright 2026, American Chemical Society. (g–i) Antifouling hydrogel coatings. (g) Hydrogel coating forms a porous diffusion barrier that allows rapid transport of small targets while excluding cells and plasma proteins. Created in BioRender. Liu, S. (2026) https://BioRender.com/v0nqlge. (h) Reduced signal degradation compared to conventional coatings. Adapted with permission [51]. Copyright 2022, Wiley‐VCH GmbH. (i) Improved in vivo signal stability with advanced hydrogel coatings. Adapted with permission [51]. Copyright 2022, Wiley‐VCH GmbH. (j–l) PEG‐functionalized nanoporous gold electrodes improve long‐term stability. (j) Nanoporous gold (npAu) coated with hyperbranched 8‐arm PEG forms hydrated, size‐selective nanochannels that accommodate aptamers while excluding large biofouling species. Adapted with permission [53]. Copyright 2025, Springer Nature. (k) SEM image of the PEG‐functionalized npAu electrode coated with 8‐arm PEG polymer. Adapted with permission [53]. Copyright 2025, Springer Nature. (l) Stable electrochemical response after implantation. Adapted with permission [53]. Copyright 2025, Springer Nature.

In ex vivo whole blood measurements, KDM reduces drift from approximately 30% to below 2% over 90 min while simultaneously improving signal amplitude and signal‐to‐noise ratio (Figure 3b). Extending to in vivo measurements in live rats, KDM‐enabled sensing allows real‐time tracking of circulating drug concentrations over 4 h across multiple dosing events, capturing dose‐dependent peaks with minimal drift throughout the experiment (Figure 3c).

Despite its effectiveness, KDM does not address the underlying physicochemical degradation of the sensor interface. Progressive loss of probe activity or surface fouling can reduce the absolute signal amplitude over time, leading to decreased signal to noise ratio (SNR) and reduced measurement precision. Although KDM can correct for shared drift components, it relies on the presence of sufficiently strong and well resolved signals at both interrogation frequencies. This requirement is not universally satisfied, as KDM relies on strong binding‐induced modulation of electron transfer kinetics, which is not observed for many aptamers, particularly those targeting proteins [100, 101]. Even when this requirement is satisfied, as degradation becomes more severe and signal levels approach the noise floor, the reliability of the differential measurement can deteriorate, ultimately limiting quantitative performance. In addition, the method relies on the assumption that drift affects both frequencies similarly, which may not always hold under dynamically changing biological conditions or when multiple degradation mechanisms are present. In addition to drift correction, accurate quantification of target concentration requires careful consideration of calibration. Downs et al. have shown that the calibration response of EAB sensors is highly sensitive to environmental factors such as temperature, media composition, and sample age [45]. Calibration curves obtained under conditions that differ from those of the measurement environment can lead to substantial errors in estimated concentration, even when drift is effectively suppressed by KDM. Subsequent work has introduced time‐resolved KDM (tKDM), which extends conventional KDM by updating the two frequencies used for KDM over time [46]. These findings highlight that achieving accurate in vivo sensing requires both improved drift‐correction strategies and carefully matched calibration conditions.

##### 3.2.1.2 Dual‐Reporter Strategy for Drift Cancellation

Ratiometric signal correction is a strategy used to mitigate baseline drift in EAB sensors by internally normalizing signal fluctuations. A foundational work by Du et al. reported a reagentless ratiometric sensor architecture that simultaneously incorporates two redox reporters, MB and ferrocene (F_C_) on a single aptamer strand [102]. In this design, MB serves as the signal reporter whose distance from the electrode changes upon target‐induced aptamer folding, while F_C_ remains stationary and functions as an internal reference. The ratio of MB to F_C_ currents thereby offers a self‐calibrating signal that compensates for variations in probe density, electrode surface area, and nonspecific degradation. Building on this concept, Li et al. extended dual‐reporter sensing in undiluted whole blood [103]. Their sensor design incorporates two redox‐active moieties, MB and anthraquinone (AQ), conjugated to distinct positions on a single aptamer strand. Upon target binding, the aptamer undergoes a conformational change that alters the electron transfer efficiency of the MB tag, while the AQ moiety remains conformationally insulated and serves as an internal reference. SWV measurements captured the ratiometric signal output, showing that the MB peak increases in a concentration‐dependent manner, while the AQ signal remains stable across different analyte levels. Using the ratiometric output, the sensor was able to track stepwise changes in kanamycin concentration in whole blood for 6 h with reduced baseline drift. While dual‐tagged aptamers offer robust drift correction, their implementation can be synthetically challenging, especially when both redox tags must be positioned at precise termini or incorporated with orthogonal chemistries. To circumvent this limitation, Zhu et al. introduced a minimalist alternative wherein only the aptamer carries a redox label (e.g., MB) and a second redox reporter (F_C_) is instead immobilized directly onto the electrode surface via a self‐assembled monolayer [104]. This spatially separated reference provides a background signal independent of aptamer folding, enabling ratiometric correction without requiring dual labeling of the probe. This design reduces synthetic complexity while maintaining the ability to normalize signal fluctuations, and has been shown to improve measurement reproducibility in buffer and serum.

These results indicate that dual‐reporter correction can improve signal robustness in complex, protein‐rich media. However, current studies are limited to in vitro conditions, and their impact on long‐term in vivo performance remains unclear. In addition, the strategy relies on the assumption that the redox peaks of the target analyte and the reporters are well separated. This imposes a significant constraint for targets that are themselves electroactive within the same potential window. For instance, the anticancer drug doxorubicin exhibits redox activity at a potential overlapping with that of the reference reporter AQ, rendering the ratiometric correction ineffective and precluding in situ monitoring using this configuration [103]. Furthermore, while MB has demonstrated robust redox performance, alternative reporters such as AQ and F_C_ may exhibit inferior stability under physiological conditions, posing further challenges for long‐term in vivo operation and limiting the generalizability of the design [105].

##### 3.2.1.3 Dual‐Aptamer Strategy for Drift Cancellation

The dual‐aptamer strategy involves the use of structurally similar aptamer pairs with differential binding affinity. Tsai et al. developed a dual‐aptamer architecture that enables internal drift correction through ratiometric electrochemical readout [101]. This approach employs two distinct aptamer probes immobilized on a single electrode surface. One is a signal‐responsive aptamer that undergoes a target‐induced conformational change, while the other is a nonresponsive reference aptamer with a similar structure but no affinity to the target molecule. Both aptamers are tagged with redox reporters, but only the target‐binding aptamer exhibits concentration‐dependent signal modulation. Similarly, differential signaling can also be achieved using an aptamer pair in which the two probes share the same sequence but carry the redox reporter at different positions, producing distinct electrochemical responses upon target binding. By computing the ratio between the two redox currents, signal drift arising from non‐specific effects such as biofouling, degradation, or environmental fluctuations can be dynamically canceled. For example, in undiluted goat serum, both channels of the AMP.P/AMP.N pair exhibited ∼22% drift over 18 h, while the ratiometric signal varied by only 0.06%. Similarly, for the ATP.P/ATP.NR pair, ∼23% signal degradation over 8 h was reduced to 0.03% after correction.

Compared to dual‐reporter approaches, the dual‐aptamer strategy avoids reliance on multiple redox chemistries, reducing the risk of peak overlap and simplifying probe design. However, several limitations remain. The identification of aptamer pairs with matched drift characteristics yet distinct target responsiveness requires labor‐intensive screening, posing challenges to their generic use. Furthermore, the method may be prone to interference when different aptamer‐drug systems are present simultaneously, as illustrated by the ambiguous signals observed with the ampicillin‐doxorubicin aptamer pair [76]. Additionally, variations in environmental parameters such as pH, ionic strength and temperature can introduce differential drift behaviors that are not fully canceled, necessitating auxiliary sensors for multi‐parametric compensation [106, 107, 108].

While this approach shows strong drift suppression in complex media, current studies are limited to in vitro conditions, and its impact on long‐term in vivo performance remains unclear. In addition, direct comparisons with established calibration methods such as KDM have not been systematically explored, leaving the relative advantages of these strategies unresolved.

#### 3.2.2 Non‐Natural Nucleic‐Acid‐Based Strategies

In contrast to calibration‐based approaches that compensate for signal drift at the data processing level, non‐natural nucleic‐acid‐based strategies aim to improve the intrinsic stability of EAB sensors by modifying the molecular structure of the aptamer itself. A major source of signal degradation in EAB sensors deployed in vivo arises from the progressive loss of functional probes at the sensor interface, which has been attributed to a combination of biofouling, monolayer instability, and, in some cases, nuclease‐mediated degradation of aptamers.

XNAs comprise a class of synthetic nucleic acid analogues in which the canonical ribose or deoxyribose sugar backbone of DNA or RNA is chemically modified or replaced with a non‐natural structure [109]. Common examples include 2’O‐methyl RNA, locked nucleic acids (LNA), peptide nucleic acids (PNA), and morpholino oligonucleotides, which differ in sugar structure, backbone composition, or both [110]. The key advantage of XNAs in biosensing applications lies in their resistance to nuclease degradation. Nucleases are evolved to recognize and cleave the natural backbone structures of DNA and RNA. By modifying this backbone, XNAs are generally not recognized as substrates by these enzymes and thus resist enzymatic cleavage (Figure 3d). This enhanced resistance allows XNA‐based aptamers to better preserve their structural integrity and sensing function in biological environments over extended periods.

Importantly, recent work has provided direct in vivo evidence that the improved molecular stability of XNAs can translate into enhanced sensor performance. Leung et al. demonstrated that replacing DNA aptamers with the OMe RNA significantly reduces in vivo signal drift in EAB sensors [48]. In a rat model, the signal from DNA‐based sensors decreased by ∼48% over 5 h of in vivo operation, whereas the corresponding XNA‐based sensors exhibited only ∼7% signal loss, representing an approximately sevenfold reduction in drift. Mechanistic analysis further revealed that, unlike in vitro conditions where fouling dominates, the in vivo drift of DNA‐based sensors is largely driven by enzymatic degradation, highlighting the importance of nuclease resistance for maintaining probe integrity in vivo.

Building on this foundation, Son et al. extended these advances by enabling continuous, seconds resolved in vivo measurements over a full week [47]. When the outputs of DNA‐ and XNA‐based sensors were reanalyzed using a common calibration curve, XNA sensors maintained a stable, near‐zero baseline over 3 days of in vivo operation, whereas DNA sensors exhibited substantial drift and noise (Figure 3e). This result highlights that the improved performance of XNA sensors arises from enhanced molecular stability and reduced signal drift, rather than affinity differences. Building on this stable baseline, the sensors were further able to continuously track circulating tobramycin concentrations across multiple intravenous dosing events, capturing dose‐dependent concentration peaks with consistent temporal resolution (Figure 3f). Notably, this extended performance was achieved without the use of protective coating or nanoengineered electrodes, highlighting that improved probe stability alone can significantly prolong sensor lifetime. Importantly, even after 1 week of continuous in vivo operation, the sensor retained approximately 40% of its initial faradaic current while still producing well‐defined voltammetric peaks, indicating preserved electrochemical functionality. Beyond reducing drift, XNA‐based sensors also exhibit improved target affinity relative to DNA‐based counterparts.

Despite these advantages, several challenges remain. The selection of functional XNA aptamers is more complex than that of natural nucleic acids and often requires specialized selection platforms and engineered polymerases [111, 112]. In addition, chemical modification of the backbone can alter binding affinity, folding dynamics, and electron transfer kinetics, necessitating re‐optimization of sensor performance [47, 48, 113]. Furthermore, only a limited number of targets currently have well‐characterized XNA aptamers, restricting the general applicability of this approach [114, 115]. An additional limitation arises for enantiomeric systems such as L‐DNA due to their reciprocal chiral specificity [116]. Because L‐DNA aptamers recognize the mirror image of the target bound by their D‐DNA counterparts, they can only directly bind native targets that are achiral, which may limit its applicability for in vivo sensing [117].

Taken together, non‐natural nucleic‐acid‐based strategies represent a powerful route toward improving the intrinsic robustness of EAB sensors. However, to date, XNA‐based stabilization has largely been explored as a standalone, probe‐level strategy, and its integration with other stability‐enhancing approaches such as antifouling coatings, or electrode interface engineering remains largely unexplored and experimentally unvalidated. Future work should therefore focus on evaluating whether combining XNA with complementary strategies can produce additive or synergistic improvements in EAB sensor lifetime, particularly for long‐term in vivo operation.

#### 3.2.3 Antifouling‐Coating‐Based Strategies

##### 3.2.3.1 Hydrogel Coatings

Hydrogel‐based coating strategies have demonstrated considerable promise in enhancing the operational stability of in vivo EAB sensors [118, 119, 120]. Their antifouling function arises primarily from the ability of hydrophilic hydrogel networks to retain large amounts of water, thereby forming a tightly bound hydration layer at the interface with surrounding tissue. This hydration shell acts as both a physical and energetic barrier, effectively preventing nonspecific protein adsorption and cellular adhesion by obstructing molecular interactions that would otherwise initiate biofouling and trigger the foreign body response (Figure 3g) [121]. Recent in vivo studies corroborated this hydration‐mediated mechanism, showing that adhesive hydrogel interfaces substantially reduced the adsorption of serum proteins such as albumin and fibrinogen, which in turn suppressed immune cell infiltration and fibrotic capsule formation around implants [122]. In addition to their hydration‐mediated antifouling effect, hydrogel coatings can also contribute size‐selective permeability. The crosslinked polymer network functions as a molecular sieve that restricts the penetration of high‐molecular‐weight species while permitting the diffusion of small‐molecule analytes to the electrode surface [123, 124, 125]. By simultaneously suppressing the access of interfering biomacromolecules and maintaining analyte transport, hydrogels help preserve the integrity of the electrode‐tissue interface. This combined action of hydration‐layer protection and selective permeability is instrumental in extending the functional lifetime of in vivo EAB sensors. Li et al. reported a hydrogel‐protected EAB sensor encapsulated with a 3 wt% agarose gel, which selectively excluded blood cells and plasma proteins but allowed rapid diffusion of target antibiotics [50]. When tested in whole blood in vitro, gel‐protected sensors maintained over 75% of their initial signal after 10 h, whereas unprotected sensors lost more than 80% of their signal under the same conditions. The hydrogel layer also protected the aptamer from nuclease degradation, as DNase I caused the unprotected sensor signal to degrade by more than 20% within 2 h while the gel‐protected sensor maintained more than 95% of its initial signal over the same period in vitro in PBS buffer. Moreover, the hydrogel protection obviated the need for algorithmic drift correction, enabling reliable real‐time tracking of sequential kanamycin administrations in vivo with high signal fidelity for 2 h. Beyond agarose, more sophisticated hydrogel chemistries have been explored to further enhance antifouling performance. For instance, Chan et al. developed a combinatorial library of non‐ionic polyacrylamide‐based hydrogels and identified a hydroxyethylacrylamide‐diethylacrylamide copolymer (F50‐C50) as a top‐performing material through high‐throughput screening [51]. When used to encapsulate a kanamycin‐responsive aptamer sensor and implanted in the femoral vein of rats for 5 days, the F50‐C50 hydrogel significantly outperformed polyethylene glycol (PEG) and uncoated controls. To evaluate the coating's antifouling stability, cyclic voltammetry (CV) was performed in ferrocyanide‐spiked blood to quantify the electrode's electrochemical responsiveness before and after implantation. The anodic peak current which reflects the accessibility of the electrode surface to redox species, serves as a standard indicator of biofouling‐induced signal degradation. CV measurements revealed that the F50‐C50 hydrogel‐coated sensors retained electrochemical responsiveness with only about 24% signal loss after 5 days in vivo, whereas most control sensors lost functionality (Figure 3h). Consistent with this, continuous in vivo measurements (Figure 3i) demonstrate that F50‐C50‐coated sensors maintain higher signal amplitude and clearer, repeatable responses to successive dosing events compared to PEG‐coated controls, which exhibit attenuated signals and progressive drift over time. These findings collectively underscore the versatility and effectiveness of hydrogel encapsulation in preserving sensor functionality under physiologically relevant conditions.

In addition to hydrogel protection, the mechanical durability of its encapsulation remains a crucial concern. Soft hydrogels are prone to swelling, deformation and delamination in dynamic tissue environments, potentially exposing the sensor surface to fouling agents and compromising long‐term functionality. To address this, Zhang et al. developed a microgel‐reinforced zwitterionic hydrogel in which densely crosslinked microgels are embedded within a softer zwitterionic matrix [126]. This architecture markedly improved both bulk mechanical strength and substrate adhesion without sacrificing the antifouling performance of the zwitterionic network. Specifically, the reinforced hydrogel exhibited nearly an order of magnitude improvement in modulus and showed enhanced retention on biomedical device surfaces under dynamic conditions. Such strategies offer a compelling route to overcoming the structural instability that has limited the chronic use of hydrogel‐protected in vivo sensors.

##### 3.2.3.2 Zwitterionic Coatings

Zwitterionic polymer interfaces have emerged as a powerful means to enhance the functional stability and host compatibility of in vivo electrochemical sensors [127]. Owing to their superhydrophilic and electrically neutral characteristics, these materials can minimize nonspecific biomolecular adsorption and attenuate immune‐driven signal disturbances [128, 129]. Unlike hydrogel coatings, where antifouling largely relies on physically entrapped water forming a hydration layer, zwitterionic polymers generate a much stronger and more persistent hydration shell through the electrostatically balanced arrangement of cationic and anionic groups along the polymer backbone. These ion pairs interact intensively with surrounding water molecules, creating an energetically favorable hydration layer that is highly resistant to protein displacement, a mechanism shown to underpin the ultralow‐fouling properties of zwitterionic hydrogels in vivo [130]. Importantly, Zhang et al. demonstrated that zwitterionic hydrogels implanted subcutaneously in mice resisted fibrotic capsule formation for months, exhibiting negligible protein adsorption and reduced macrophage recruitment compared with PEG or conventional hydrogels [131]. As a result, zwitterionic interfaces exhibit exceptionally low protein adsorption and greatly diminished immune cell recruitment, thereby preventing fibrotic encapsulation and enabling significantly longer functional lifetimes for in vivo EAB sensors. In a representative study, Xie et al. engineered a zwitterionic phosphorylcholine‐based polymer (poly(MPC)) and immobilized it onto commercial continuous glucose monitors (CGMs) via a polydopamine (PDA) mediated surface modification process [132]. Specifically, a thin PDA film was first deposited onto the electrode by spontaneous oxidative polymerization [133], followed by covalent attachment of thiol‐functionalized poly(MPC) via Michael addition. In both rodent and non‐human primate models, poly(MPC)‐coated sensors triggered markedly lower inflammatory responses, evidenced by live‐animal fluorescence imaging and transcriptional profiling and delivered accurate glucose readings without requiring frequent recalibration. By contrast, unmodified sensors experienced substantial signal instability and deviation from reference values during the initial days post‐implantation. This coating strategy thus not only enhances measurement fidelity but also alleviates the need for burdensome manual correction, an important factor in clinical usability.

Building on a similar PDA‐anchoring concept, Feng et al. employed sulfobetaine methacrylate (SBMA) to construct a zwitterionic SBMA‐PDA interface on carbon nanotube modified carbon fiber microelectrodes for in vivo ascorbic acid monitoring in rat brain [134]. The PDA primer provided strong adhesion to the electrode surface and enabled covalent SBMA grafting via Michael addition, yielding an ultrathin, highly hydrophilic and Reactive Oxygen Species (ROS)‐resistant antifouling layer [135]. In both healthy and Parkinson's disease rat models, the SBMA‐PDA‐coated sensors maintained 92% of their initial sensitivity after 2 h of implantation, while inducing minimal acute neuroinflammatory responses. The antioxidant properties of PDA further contributed to coating stability under oxidative stress, highlighting the robustness of this approach for neurochemical sensing in complex pathological environments.

More recently, GhavamiNejad et al. demonstrated a complementary approach using a carboxybetaine‐based zwitterionic hydrogel microneedle (MCB‐HE MN) patch integrated with a molecular pendulum (MP) electrochemical sensor for continuous protein detection in vivo [136]. Here, the zwitterionic hydrogel served not only as an antifouling interface but also as a protective matrix for antibody‐based recognition elements, preserving their secondary structure through UV‐induced crosslinking and γ‐irradiation sterilization‐processes often essential for clinical translation. Molecular dynamics simulations revealed that multiple non‐covalent interactions (hydrogen bonds, salt bridges, π‐cation interactions) between the MCB‐HE and the antibody reduced structural fluctuations, lowered solvent‐accessible surface area and enhanced rigidity, thereby improving resilience under prolonged physiological exposure. Functionally, the hydrogel maintained F_C_ redox tag stability for at least 30 h in simulated interstitial fluid and preserved sensing performance after repeated mechanical deformation. In streptozotocin‐induced diabetic rats, the integrated device provided real‐time insulin tracking for up to 2 h, with electrochemical signals closely matching ELISA measurements in both plasma and interstitial fluid, even under circadian rhythm disruption that altered insulin distribution kinetics.

Beyond enzyme and antibody‐based in vivo biosensors, zwitterionic coatings have also been extensively employed to enhance the antifouling properties of EAB sensors [44]. For example, Ding et al. developed a PDA‐APDMAO copolymer coating that forms a robust hydration barrier, enabling rapid aptamer coupling and long‐term resistance to serum fouling [137]. Wu et al. demonstrated that a poly(sulfobetaine methacrylate) (PSB) coating on flexible microelectrode arrays protected cocaine aptasensors from albumin adsorption and DNase‐1 degradation, preserving in vivo signal fidelity during repeated brain infusions [98]. More recently, Song et al. designed a zwitterionic peptide with shortened carboxyl‐amino spacing, which exhibited superhydrophilicity, enhanced rigidity and protease resistance. When integrated with aptamers, the peptide‐based interface maintained antifouling performance in undiluted serum for over 3 weeks [138]. McGowan et al. employed short‐chain carboxybetaine and sulfobetaine monolayers on nanoporous electrodes, achieving superior antifouling in whole serum compared to conventional mercaptohexanol passivation, while retaining aptamer sensitivity [139]. Most recently, Duan et al. further advanced this concept by developing an integrated SBMA@PDA zwitterionic copolymer coating that simultaneously provides strong surface adhesion and a dense hydration layer [52]. The coating leverages the oxidative self‐polymerization of dopamine to anchor poly(sulfobetaine methacrylate) chains on the electrode, forming a uniform hydrophilic interface that resists protein adsorption while preserving rapid electron transfer between MB reporters and the electrode. To assess stability under tissue‐like conditions, they incorporated the coating into a microneedle‐based EAB sensor for continuous in situ vancomycin detection. In a BSA‐spiked artificial interstitial‐fluid phantom gel, the SBMA@PDA‐coated microneedle sensor exhibited less than 6% signal drift during 5 h of continuous operation, compared with 11% for the uncoated device. When inserted into porcine skin, the coated sensor maintained 91% of its initial signal after 3 h, while uncoated electrodes lost over 25% and displayed elevated noise. These findings confirm that the zwitterionic hydration layer effectively suppresses fouling from proteins and cells, even in mechanically stressed and biologically complex environments. Collectively, these studies not only highlight the effectiveness of zwitterionic chemistries in EAB sensors against nonspecific adsorption and enzymatic degradation but also underscore the extensibility of zwitterionic antifouling strategies across diverse in vivo biosensor designs.

Nevertheless, important limitations remain. For the poly(MPC)‐coated CGMs, the anti‐inflammatory benefits mainly address acute‐phase host responses and have yet to demonstrate protection against other degradation mechanisms such as enzymatic cleavage of affinity probes or nanoscale fouling layer accumulation. For the MCB‐HE MN platform, although it preserves antibody functionality through sterilization and mechanical stress, the reported continuous monitoring duration in vivo was limited to 2 h and long‐term operational stability over days to weeks remains to be established.

#### 3.2.4 Nanoengineering‐Based Strategies

##### 3.2.4.1 Au‐Based Nanostructures

Nanoporous gold (npAu) electrodes were originally introduced to address the signal loss and decreased sensitivity associated with miniaturized electrochemical sensors [140]. By increasing the electroactive surface area and enhancing electron transfer efficiency, these nanostructured interfaces helped mitigate diffusion limitation and signal loss of microscale devices [55, 141, 142]. Such architectures have also been repurposed for improving sensor longevity under biofouling‐prone conditions [143, 144]. Seo et al. demonstrated that nanoporous electrodes could serve as passive antifouling barriers by physically shielding aptamer probes from large fouling species while maintaining access to small‐molecule targets [17]. As shown in the Scanning Electron Microscope (SEM) image, the nanoporous gold electrode features a bicontinuous 3D nanostructure that forms confined spaces where aptamer probes and redox reporters reside. This architecture was directly compared to a conventional planar electrode in terms of signal stability and responsiveness in undiluted serum. Over a 16‐h incubation period, the nanoporous sensor retained over 80% of its baseline electrochemical signal, whereas the planar sensor experienced continuous signal degradation. In addition, the target‐induced signal gain of the planar sensor was almost completely suppressed after fouling, while the nanoporous sensor preserved its sensing performance, showing minimal loss in responsiveness. These results highlight the dual role of npAu, which not only enables device miniaturization and signal amplification but also provides a structurally intrinsic antifouling mechanism that significantly extends sensor functionality in complex biological environments.

Chen et al. further integrates npAu with a hyperbranched PEG coating to protect EAB sensors from biofouling, degradation and signal drift in complex biological environments (Figure 3j) [53]. The design draws inspiration from the intestinal mucosa, where a dense glycocalyx coating atop epithelial microvilli creates a spatially restricted zone that enables selective molecular transport while shielding receptors from interfering biomolecules. The sensor features a npAu scaffold that hosts redox‐tagged aptamer switches within internal nanocavities, further insulated by a hyperbranched PEG coating. Figure 3k shows an SEM cross‐section of the PEG‐coated nanoporous gold interface, illustrating how the hyperbranched polymer layer conformally fills and lines the interconnected pores to create a hydrated, size‐selective nanoenvironment that insulates the underlying aptamer layer. This hierarchical structure allows small‐molecule targets to reach aptamer binding sites while excluding nucleases and serum proteins, effectively minimizing nonspecific interactions and maintaining signal integrity over time. Electrochemical characterization confirmed the robustness of this strategy. SWV measurements showed minimal baseline drift after 28 days of continuous exposure to human serum in vitro, retaining over 70% of the initial signal. Notably, the voltammograms remained well‐defined, demonstrating preserved aptamer functionality. Furthermore, the sensor maintained dose‐dependent responses to kanamycin when interrogated at discrete time points (days 1, 4, and 7) following intravenous implantation in freely moving rats, with signal gain curves showing consistent reversibility and sensitivity (Figure 3l). However, the sensors were surgically implanted within rat femoral veins for multiple days and subsequently extracted for evaluation, indicating that their performance was assessed post‐implantation rather than through continuous, real‐time monitoring in vivo.

These results highlight the effectiveness of integrating nanostructured electrodes with polymer‐based antifouling coatings to create a robust sensing interface. The sensor enables long‐term monitoring, achieving up to 28 days of stability in human serum in vitro and retaining functionality after up to 7 days of implantation in freely moving animals. However, this design also imposes a molecular size cutoff. While it effectively excludes interferents, it simultaneously limits analyte accessibility, making it best suited for small molecules (<1 kDa) and less applicable to larger targets such as peptides and proteins. Future efforts may not only explore tunable pore architectures or dynamic coatings to expand the range of detectable targets while preserving long‐term stability, but also incorporate continuous in vivo evaluation to validate long‐term operational performance under real‐time sensing conditions.

Recent work has also investigated nanoporous gold electrodes with modified structural characteristics under both in vitro and in vivo conditions. Zhu et al. reported a nanoporous gold architecture fabricated via a stress‐mitigated dealloying process, designed to enhance structural integrity and reduce degradation during prolonged operation [46]. In vitro evaluation in undiluted fetal bovine serum showed that the sensor maintained approximately 80% of initial signal over 9 days. In vivo measurements further demonstrated the ability to perform longitudinal pharmacokinetic monitoring in rats over 6 days, during which target‐dependent signals remained detectable across repeated dosing cycles. In this work, signal stability is supported by the combined use of nanoporous electrode design and time‐resolved kinetic differential measurement (tKDM), a calibration strategy that extends dual‐frequency interrogation by incorporating time‐dependent signal evolution to correct drift during continuous measurements. As such, the multi‐day in vivo performance cannot be attributed to electrode nanostructuring alone, but reflects the combined contributions of both structural and signal‐processing approaches.

##### 3.2.4.2 Carbon‐Based Structures

The fabrication of high‐fidelity nanoporous gold structures typically requires multi‐step processing involving metal dealloying and careful structural tuning, which can be technically demanding and costly [145, 146]. Alternatively, carbon‐based nanostructures offer a simpler and more scalable platform. For instance, laser‐induced graphene (LIG) electrodes can be patterned directly onto flexible substrates under ambient conditions via CO_2_ laser engraving, yielding a three‐dimensional, nanostructured porous morphology with high surface area [147, 148] and demonstrated electrochemical performance. Their electrochemical applicability has been validated in wearable biosensors [149, 150] and multiple studies have shown that LIG can be functionalized with aptamers for subsequent electrochemical detection of target analytes in complex media [151, 152]. Fenzl et al. demonstrated that laser‐scribed graphene could be modified via π–π stacking of 1‐pyrenebutyric acid followed by covalent coupling of amino‐functionalized thrombin aptamers [54]. Notably, the sensing mechanism in this system relies on diffusion‐limited access of an external redox probe rather than conformational switching of a redox‐labeled aptamer and therefore does not fall within the classical EAB sensing paradigm. Nevertheless, such findings highlight the promise of LIG as a scalable carbon‐based platform for biosensing applications. Similarly, other carbon‐based nanostructured electrodes such as those constructed from carbon nanotubes (CNTs) or carbon black can be engineered into nanoporous architectures with tunable surface morphology, providing large electroactive surface area, high electrical conductivity and versatile surface chemistry for biomolecule immobilization [153, 154, 155]. However, it is important to emphasize that there is currently no direct evidence demonstrating that carbon‐based electrodes improve in vivo operational stability or duration in EAB sensors. Their antifouling behavior and long‐term performance under in vivo conditions remain largely unexplored and require systematic evaluation, particularly in comparison with established gold‐based systems.

#### 3.2.5 Advanced‐Immobilization‐Based Strategies

Traditional immobilization strategies based on thiol‐based SAMs are prone to degradation under complex biological conditions and frequent electrochemical interrogation [156]. To overcome this limitation, various alternative immobilization methods that do not rely on SAMs have been developed. Among these alternatives, carbon‐based electrodes have gained considerable attention as a versatile platform for stable aptamer immobilization [157]. Carbon electrodes offer several key advantages, including high electrical conductivity, large surface area and the presence of reactive surface functional groups such as hydroxyl and carboxyl groups, which can be introduced or enhanced through mechanical or electrochemical treatment [158] and these properties enable both covalent and non‐covalent aptamer attachment.

##### 3.2.5.1 Electrografting

Electrografting has emerged as a promising approach due to its versatility and ability to form strong covalent linkages [159]. Several studies have explored electrografting‐based strategies for aptamer immobilization [160, 161], some of which also employed electrochemical methods for sensor characterization [162, 163]. In a representative study, Pellitero and Arroyo‐Currás demonstrated that the electrografting of primary aliphatic amines, specifically aminohexane, onto glassy carbon electrodes enables the formation of densely packed, covalently bonded monolayers suitable for aptamer immobilization (Figure 4a) [56]. The resulting C‐N bond‐based monolayers exhibit significantly improved stability over conventional thiol‐gold assemblies, particularly when electrochemical interrogation is conducted in a positive voltage window (0.2–0.7 V vs Ag/AgCl). In comparison, electrografted aminohexane monolayers on carbon maintained stable capacitive currents for 48 h of continuous cycling, whereas thiol‐based monolayers on gold showed marked signal degradation under the same conditions (Figure 4b,c). Moreover, this enhanced stability was preserved in undiluted human serum, indicating strong resistance to thiol‐exchange reactions and biofouling. These findings underscore the potential of carbon‐based aptamer sensors prepared via electrografting as a robust alternative to thiol‐on‐gold architectures for long‐term electrochemical biosensing applications.

**FIGURE 4:**
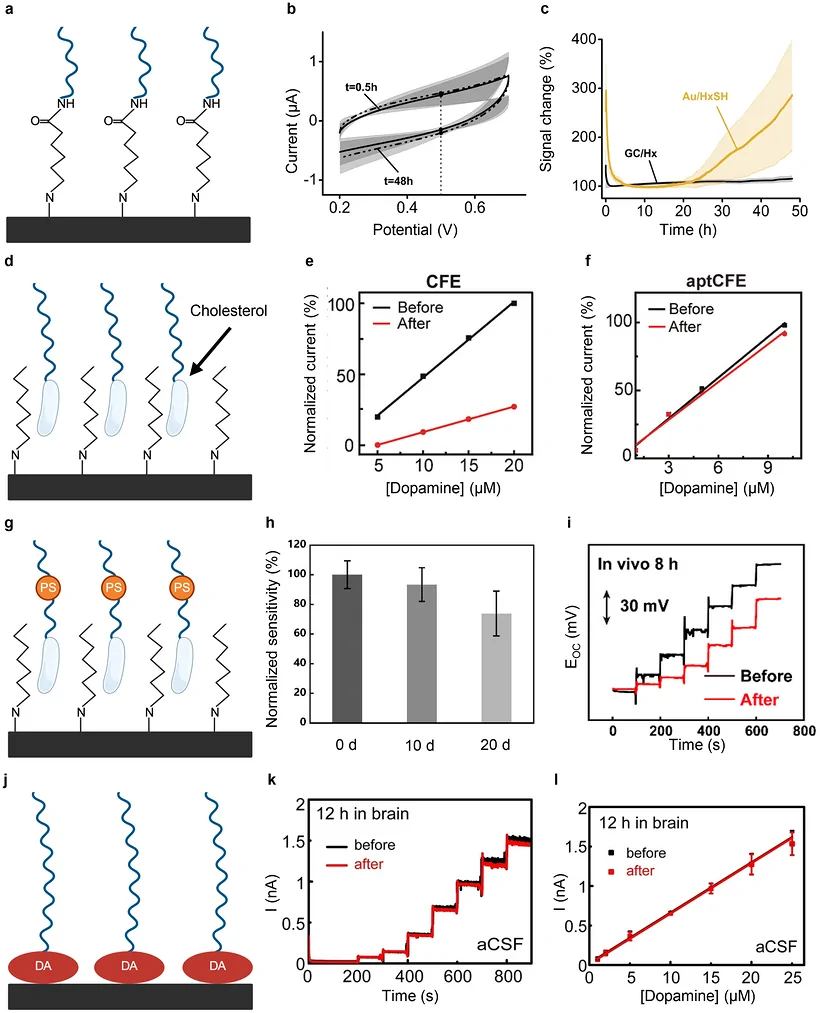
Advanced immobilization strategies for EAB sensors. (a–c) Electrografted molecular layers enhance stability against electrochemical desorption. (a) Primary aliphatic amines are electrografted onto carbon electrodes to form covalent C‐N bonds, creating a stable anchoring layer for aptamers. Created in BioRender. Liu, S. (2026) https://BioRender.com/pgj3193. (b) Electrografted monolayers on glassy carbon display minimal peak‐current loss after prolonged cycling, even at oxidative potentials, demonstrating strong resistance to voltage‐induced cleavage. Reproduced under the terms of the CC‐BY license [56]. Copyright 2022, Springer Nature. (c) Long‐term amperometric interrogation shows rapid degradation of thiol‐gold linkages, whereas covalently grafted C‐N layers maintain signal fidelity, highlighting the enhanced stability of carbon‐based covalent immobilization. Reproduced under the terms of the CC‐BY license [56]. Copyright 2022, Springer Nature. (d–f) Supramolecular cholesterol‐aptamer assemblies provide noncovalent yet stable hydrophobic anchoring. (d) A cholesterol‐modified aptamer self‐assembles into a hydrophobic alkyl layer on carbon fiber electrodes (CFE) pre‐modified with hexylamine, forming a bio‐selective supramolecular interface. Created in BioRender. Liu, S. (2026) https://BioRender.com/pgj3193. (e) Bare CFE show substantial signal degradation and reduced dopamine sensitivity following implantation. Reproduced with permission [57]. Copyright 2020, Wiley‐VCH GmbH. (f) Cholesterol‐anchored aptamer‐modified CFE maintain a linear response to dopamine after implantation. Reproduced with permission [57]. Copyright 2020, Wiley‐VCH GmbH. (g–i) Phosphorothioate (PS) modifications protect aptamers from nuclease degradation. (g) Incorporating PS backbones on the cholesterol‐aptamer interface improves nuclease resistance via steric hindrance and sulfur‐mediated stabilization. Created in BioRender. Liu, S. (2026) https://BioRender.com/pgj3193. (h) Quantitative measurements show enhanced sensor sensitivity preserved over 20 days, indicating long‐term biochemical integrity under biologically relevant conditions. Reproduced with permission [58]. Copyright 2024, American Chemical Society. (i) Dopamine recordings remain stable after 8 h in vivo, indicating reversible sensing and preserved interface integrity. Reproduced with permission [58]. Copyright 2024, American Chemical Society. (j–l) Thiol‐Michael addition enables rapid, reagent‐free covalent immobilization for in vivo sensing. (j) Schematic illustration of aptamers covalently immobilized on the electrode surface through thiol‐Michael addition chemistry. Created in BioRender. Liu, S. (2026) https://BioRender.com/pgj3193. (k) Stepwise amperometric responses measured before and after a 12 h brain implantation show nearly identical current steps, indicating preserved sensor function. Reproduced with permission [59]. Copyright 2022, Wiley‐VCH GmbH. (l) Calibration curves collected in artificial cerebrospinal fluid (aCSF) demonstrate that dopamine dose‐response behavior remains linear and almost unchanged after implantation, confirming that thiol‐Michael linkages maintain probe stability against multiple signal degradation pathways. Reproduced with permission [59]. Copyright 2022, Wiley‐VCH GmbH.

Beyond covalent bonding strategies, non‐covalent anchoring methods have also been explored to improve the operational stability of aptamer‐based sensors. One such approach involves the use of cholesterol‐modified aptamers assembled onto carbon fiber electrodes pretreated with electrochemically grafted alkyl chains [57]. This strategy leverages hydrophobic interactions between the cholesterol moiety and the alkyl monolayer to achieve stable, non‐covalent aptamer anchoring (Figure 4d). The electrochemically grafted alkyl layer provides a robust and hydrophobic interface, which not only facilitates aptamer assembly but also helps resist desorption and nonspecific adsorption under physiological conditions. Such supramolecular assemblies have been shown to enable highly selective and sensitive detection of dopamine in vivo. Notably, aptamer‐functionalized electrodes retained their sensing performance following implantation in the rat brain for 2 h, whereas unmodified carbon electrodes exhibited a threefold signal loss under the same conditions (Figure 4e,f). These results suggest that the aptamer layer, together with the hydrophobic anchoring interface, provides anti‐fouling properties that help preserve electrode functionality in complex biological environments.

Building on this strategy, Ni et al. introduced an additional layer of stabilization by modifying the cholesterol‐anchored aptamers with phosphorothioate (PS) backbones (Figure 4g) [58]. While the core anchoring mechanism remained consistent with the approach reported by Li et al., the incorporation of PS modifications enhanced resistance to nuclease‐mediated degradation. This refinement led to improved biostability of the resulting galvanic redox potentiometric (aptGRP) sensors. Compared to previous systems, the aptGRP sensors preserved about 90% of sensitivity after 10 days of storage in PBS (Figure 4h) and retained high sensitivity after exposure to fetal bovine serum, exhibited minimal signal degradation after 8 h of brain implantation (Figure 4i). These findings demonstrate that combining supramolecular anchoring with backbone‐stabilized aptamers can further improve the robustness and longevity of in vivo aptamer‐based sensing platforms.

##### 3.2.5.2 Thiol‐Michael Addition

The thiol‐Michael addition provides a robust approach to form stable covalent linkages between thiol‐modified aptamers and carbon electrodes. In this reaction, thiols attack electron‐deficient conjugated groups (e.g., quinones, acrylates, maleimides), producing strong thioether bonds that exhibit substantially greater resistance to cleavage than conventional thiol‐gold coordination [164]. Li et al. introduced a purely electrochemical strategy to generate covalent aptamer‐electrode interfaces using dopamine (DA) as a multifunctional linker (Figure 4j) [59]. The process begins with electrochemical oxidation of DA (from +0.8 to +1.6 V) to form a thin quinone‐rich layer on the CFE surface. Subsequently, thiol‐modified dopamine aptamers (HS‐DA‐apt) are immobilized via nucleophilic addition under a controlled potential of +0.55 V, achieving dense and uniform probe coverage within 5 min. Unlike conventional thiol‐gold chemistry or noncovalent adsorption, this approach eliminates the need for pre‐functionalized surfaces and prolonged incubation steps, while simultaneously enhancing attachment stability and minimizing nonspecific adsorption.

To evaluate in vivo performance, the sensor was implanted into the rat striatum and tested before and after 12 h. As shown in Figure 4k,l, the amperometric responses to dopamine remained consistent and calibration curves showed negligible deviation, indicating excellent short‐term operational stability. In contrast to previously reported noncovalent sensors, which lost function within hours, this covalent architecture resisted both protein fouling and thiol exchange. These results underscore the robustness of covalent aptamer‐carbon interfaces for continuous neurochemical monitoring ex vivo.

Despite these advances, certain limitations remain. Although the dopamine‐derived monolayer permits rapid electron transfer, it may introduce potential interference unless adequately passivated [165]. Furthermore, while the short‐term stability is impressive, the long‐term viability under chronic implantation conditions especially involving immune responses and tissue remodeling, warrants further investigation. Future developments may include integration with antifouling materials or multifunctional surface chemistries to extend operational lifetimes and broaden target applicability [166].

Taken together, these strategies differ substantially in their level of experimental validation and relevance to long‐term in vivo operation. At the highest level, approaches supported by direct in vivo evidence over extended timescales, most notably non‐natural nucleic‐acid‐based combined with KDM calibration [47] and nanoengineering‐based strategies incorporating antifouling coatings [53], currently provide the most compelling demonstrations of prolonged functionality in vivo. In comparison, antifouling‐coating‐based strategies, including hydrogel and zwitterionic interfaces [50], as well as covalent immobilization approaches [166], have shown clear improvements in stability and signal retention. However, existing in vivo studies are generally limited in duration or scope, and their ability to sustain long‐term continuous operation in vivo remains to be established. By contrast, calibration‐based methods, such as dual‐reporter and dual‐aptamer strategies [101, 102, 103, 104], have primarily been demonstrated in vitro or under partially relevant conditions. While effective at correcting signal drift, these approaches do not address the underlying physicochemical degradation processes and lack systematic long‐term in vivo validation. As such, their translational potential for continuous in vivo sensing remains uncertain.

## 4 Challenges and Advances in System Integration

### 4.1 Electronics for In Vivo EAB Sensors

Electronic modules are the backbone of in vivo EAB sensors, providing the essential functions of power management, signal generation and acquisition, data processing and wireless communication [167]. Their design dictates not only the fidelity of electrochemical readout but also the overall miniaturization, biocompatibility and long‐term stability of the sensing platform in vivo [168]. Recent advances in ultralow‐power integrated circuits and wireless communications have converged to push these systems from proof‐of‐concept demonstrations toward clinically viable devices.

#### 4.1.1 Front‐End Circuitry

The front‐end of in vivo EAB sensors is tasked with generating stable excitation signals and faithfully recording the resulting electrochemical responses. Depending on the sensing modality, these responses may manifest as currents (amperometric sensing), voltages (potentiometric sensing), or voltage‐current curves (voltammetric sensing). The ability of the front‐end to maintain low noise and low power consumption is therefore central to the accuracy and longevity of the system.

In early demonstrations, researchers developed potentiostat circuits tailored to their sensing modality. These designs, often built on discrete operational amplifiers and analog switches, allowed fine‐grained control over electrode biasing schemes and signal conditioning [169]. For example, Ainla et al. developed an open‐source wireless potentiostat that interfaced directly with smartphones via Bluetooth, demonstrating low‐cost, flexible deployment of electrochemical assays in portable or implantable formats [170]. Similarly, other works have presented potentiostat architectures optimized for amperometric or voltammetric measurements, emphasizing circuit simplicity to minimize area and energy overhead [62, 93, 171]. While these approaches offer adaptability, they often face limitations in scalability and reproducibility.

To overcome these challenges, commercialized electrochemical front‐end chips have been introduced, providing standardized and compact solutions. A representative early device is the Texas Instruments LMP91000, one of the first programmable analog front‐ends for electrochemical sensing. It integrates a transimpedance amplifier, programmable bias generator and gain settings within a small package, supporting three‐electrode configurations for amperometric and potentiometric sensing. The LMP91000 was widely used in portable and wearable biosensors, though its capabilities were limited to basic electroanalytical techniques [172, 173].

More recently, application‐specific integrated circuits (ASICs) for electrochemical measurements have gained traction, offering highly integrated, validated and power‐efficient solutions. A representative example is the Analog Devices AD5940, a precision analog front‐end designed for a wide range of electrochemical modalities. Beyond conventional amperometric and impedance measurements, the AD5940 enables advanced voltammetric techniques such as SWV and DPV, which are essential for aptamer‐based biosensors and for resolving small redox currents in complex biological environments. It integrates a programmable transimpedance amplifier, waveform generator, low‐noise ADC and flexible sensor interfaces, significantly reducing system complexity. By combining electrochemical excitation and readout functions in a single chip, it enables reliable and miniaturized front‐ends for implantable devices. Reports have demonstrated its use in continuous glucose monitoring and aptamer‐based biosensors, showing high sensitivity and reduced design overhead compared with discrete potentiostats [60, 61].

#### 4.1.2 Wireless Communication

Wireless communication is indispensable for in vivo EAB sensors, enabling real‐time monitoring without percutaneous connections that increase infection risk [174]. A range of microcontrollers and system‐on‐chip (SoC) platforms have been leveraged to support ultralow‐power signal processing and wireless transmission, with STM8/STM32, Cypress CYBLE modules and Nordic Semiconductor nRF52 series representing the most reported solutions.

The STM8 (8‐bit) and STM32 (32‐bit ARM Cortex‐M) families from STMicroelectronics have been widely adopted for implantable and wearable sensing systems owing to their robust peripheral support, cost‐effectiveness, and scalability [63, 172, 175, 176]. Particularly STM32 devices offer higher computational capacity, integrated analog‐to‐digital converters, and real‐time control, making them suitable for concurrent management of electrochemical signal acquisition and wireless communication stacks. Their relatively low standby power consumption and availability of ultralow‐power variants (e.g., STM32L series) improve their feasibility for chronic implantable devices, though additional RF front‐ends are typically required.

Cypress CYBLE modules integrate a Bluetooth Low Energy (BLE) radio with a programmable microcontroller. These modules have been reported in point‐of‐care and early implantable prototypes for their turnkey BLE connectivity, compact form factor and simplified development environment [60, 61, 150, 177]. Their embedded radio stack reduces design overhead, enabling rapid prototyping. However, compared with Nordic SoCs, CYBLE modules often exhibit higher active power consumption, which may limit their use in long‐term untethered implants unless paired with advanced power management strategies or intermittent communication schemes.

The Nordic nRF52832 BLE SoC has become a benchmark solution in recent implantable device studies [178, 179, 180, 181]. Built on an ARM Cortex‐M4 core with a highly integrated 2.4 GHz transceiver, it provides a favorable balance between computational power, low‐energy operation and wireless throughput. The device includes hardware acceleration for digital signal processing, multiple SPI/I^2^C/UART interfaces for sensor front‐ends and an optimized BLE stack. Its demonstrated use in wireless electrochemical potentiostats and implantable organ interfaces underscores its feasibility for miniaturized implants, with active currents as low as 7.07 mA during transmission [68].

The nRF52840, an advanced version in the nRF52 family, expands wireless capabilities to Bluetooth 5, Thread and Zigbee while offering increased RAM (256 kB) and flash memory (1 MB). These enhancements make it attractive for complex, multi‐sensor implantable systems requiring robust data handling and longer‐range wireless communication. While its power draw is moderately higher than the nRF52832, the chip's advanced sleep modes and dynamic power management preserve suitability for low‐duty‐cycle implants. Its extended protocol support also opens avenues for interoperability in multi‐device monitoring networks [182].

### 4.2 Fully Integrated In Vivo EAB Sensors

Efforts toward fully integrated EAB sensing systems where EAB sensors, electrochemical readout, wireless communication and power management coexist within a single platform have accelerated in recent years. A recent pilot first‐in‐human study reported a minimally invasive EAB patch for continuous vancomycin monitoring in dermal interstitial fluid, establishing that fully integrated in vivo EAB sensing is now experimentally feasible in a human setting [183]. In that work, fine‐needle EAB sensors and the supporting electronics for voltammetric interrogation and onboard data storage were incorporated into a single skin‐mounted patch, enabling 5 min‐resolved measurements for over 24 h, although signal degradation limited the highest‐quality analysis primarily to the first 12 h. While this study represents an important milestone, it provides only limited system‐level detail regarding the underlying electronics architecture, wireless functionality, and power management strategy. To better understand how such systems can be realized and extended toward prolonged in vivo operation, it is therefore instructive to consider a broader set of bioelectronic platforms that, although not always EAB‐based, demonstrate key principles of system‐level integration under physiological conditions. Collectively, these studies establish a technological foundation for the development of fully integrated EAB sensors capable of longitudinal in vivo monitoring.

For wearable applications, Ye et al. reported a fully integrated wireless aptamer‐based nanobiosensor that illustrates how biorecognition, microfluidics and electronics can be seamlessly combined into a single skin‐interfaced patch (Figure 5a) [60]. The device autonomously induced sweat via iontophoresis, guided precise sampling with capillary bursting valves and performed in situ electrochemical analysis coupled with real‐time calibration using multiplexed pH, ionic strength, and temperature sensors (Figure 5b). These fluidic and sensing elements were directly interfaced with a flexible printed circuit board (PCB) that integrated the electrochemical front end, signal processing unit and Bluetooth wireless communication, powered by a rechargeable coin cell (Figure 5c). Quantitatively, the sensor achieved an ultra‐low LOD of 0.14 pM for estradiol in sweat and captured cyclical hormone fluctuations correlating strongly with serum levels. This work exemplifies how aptamer‐based sensing chemistry can be embedded within a complete wearable electronic architecture for real‐time molecular monitoring.

**FIGURE 5:**
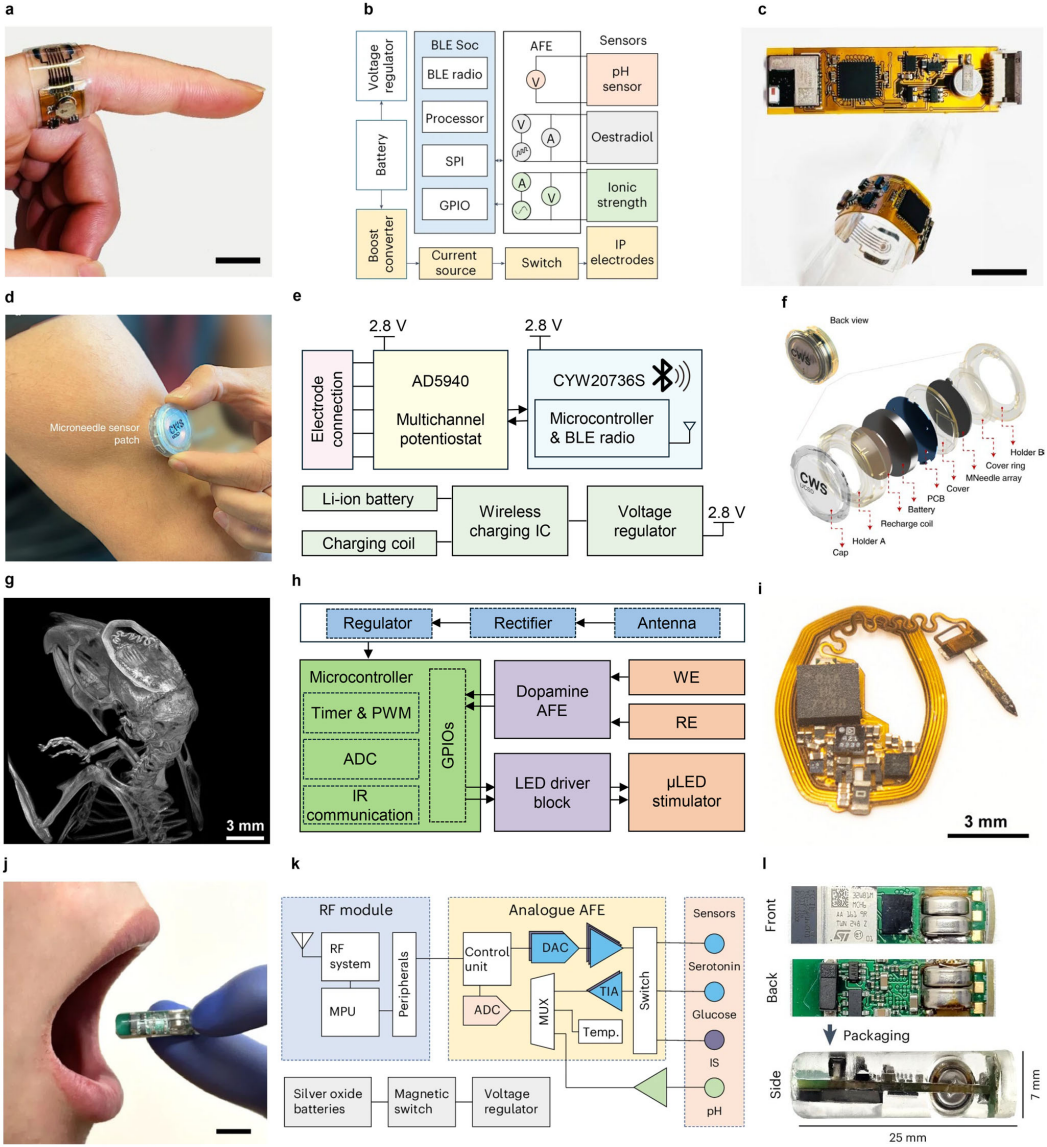
System‐level integration of wireless wearable and implantable devices for continuous biochemical sensing. (a–c) Wireless multimodal wearable platform integrating EAB sensors. (a) Flexible skin‐mounted system incorporating pH, oestradiol, and ionic‐strength aptamer sensors for multiplex biochemical monitoring. Reproduced with permission [60]. Copyright 2024, Springer Nature. (b) Block diagram of integrated electronics, including BLE SoC, analog front‐end (AFE), multisensor inputs, current sources, switching networks and on‐board processing. Reproduced with permission [60]. Copyright 2024, Springer Nature. (c) Fabricated device showing flexible architecture that couples sensors with wireless electronics. Reproduced with permission [60]. Copyright 2024, Springer Nature. (d–f) Microneedle‐based wireless interstitial fluid sensing patch. (d) Microneedle array patch for minimally invasive in situ molecular sensing. Reproduced with permission [61]. Copyright 2022, Springer Nature. (e) System architecture featuring a multichannel potentiostat (AD5940), BLE microcontroller, Li‐ion battery, wireless charging unit, and voltage regulation. Adapted with permission [61]. Copyright 2022, Springer Nature. (f) Exploded view showing sensor‐housing assembly, microneedle array, printed circuit board (PCB), coil and battery enabling biochemical readout through interstitial fluid. Reproduced with permission [61]. Copyright 2022, Springer Nature. (g–i) Fully implantable wireless device for neurochemical monitoring. (g) Micro‐CT image of the implanted flexible neurochemical sensing device. Reproduced with permission [62]. Copyright 2022, American Chemical Society. (h) System‐level block diagram illustrating the power module, microcontroller sub and peripheral modules including the dopamine sensor and µLED stimulator. (i) Photograph of the miniaturized, coiled implant incorporating the power harvesting coil, integrated electronics and encapsulated components designed for long‐term implantation. Reproduced with permission [62]. Copyright 2022, American Chemical Society. (j–l) Ingestible electrochemical capsule for in vivo gastrointestinal sensing. (j) The ingestible capsule is positioned orally prior to deployment in the gastrointestinal tract. Reproduced with permission [63]. Copyright 2025, Springer Nature. (k) The integrated circuit architecture includes RF readout, ADC/DAC modules, transimpedance amplifiers, magnetic switching, temperature compensation and multi‐analyte sensing channels for serotonin, glucose, pH and indoxyl sulfate. Reproduced with permission [63]. Copyright 2025, Springer Nature. (l) The fully packaged capsule incorporates an Ag/AgCl reference electrode, microelectrodes, silver oxide batteries and waterproof encapsulation, enabling multi‐hour molecular sensing in gastric environments. Reproduced with permission [63]. Copyright 2025, Springer Nature.

For in vivo applications, Tehrani et al. developed semi‐implantable, enzyme‐based electrochemical biosensors for monitoring metabolites in interstitial fluid (Figure 5d) [61]. Polymeric microneedles, fabricated by hot embossing and coated with conductive carbon electrodes, penetrated the epidermis to access interstitial fluid. These microneedles formed the sensing interface, which was subsequently wired to a flexible PCB hosting potentiostat circuitry and Bluetooth communication (Figure 5e,f). While the sensing relied on catalyst‐based reactions that differ from EAB sensors, the modular design highlights a potential integration pathway through minimally invasive microneedles for in vivo access and externalized electronics for system‐level functionality.

Stuart et al. further demonstrated a fully implantable platform that combined electrochemical sensing with optogenetic stimulation in freely moving mice (Figure 5g) [62]. Here, the biorecognition interface consisted of a SWCNT‐carbon fiber electrode for catecholamine detection, which was integrated with a µLED probe for neural stimulation. Signal transduction was managed by an onboard analog front end, while energy and wireless communication were supported by a center‐tapped resonant antenna that enabled dual‐polarity power harvesting (Figure 5h). By eliminating batteries, the system reduced system bulk and enabled a full implantable form factor (Figure 5i), representing another design pathway for fully integrated EAB sensors.

Min et al. demonstrated a fully integrated EAB sensor for metabolic profiling in the gut, though long‐term monitoring for more than a day remains to be demonstrated [63] (Figure 5j). The capsule incorporates a canonical folding‐based serotonin EAB sensor, in which an MB‐tagged aptamer transduces target binding into an electrochemical signal. This sensing interface is integrated with an AD5940‐based analog front end for signal readout, a low‐power Bluetooth module for wireless data transmission, and silver oxide microbatteries for onboard power (Figure 5k). All components are encapsulated within a biocompatible housing that enables stable performance in the gastrointestinal environment (Figure 5l). In vivo experiments in rabbits demonstrated continuous, reagentless, and reversible monitoring of serotonin, along with glucose, pH, ionic strength and temperature, for more than 20 h. To mitigate biofouling, the system employs a Nafion coating, which provides partial protection through size exclusion and electrostatic repulsion. However, such strategies are typically insufficient to prevent protein adsorption and signal degradation over extended in vivo timescales. Consistent with this limitation, challenges related to probe degradation and biofouling remain to be addressed to enable longer‐term functionality. Together, these studies highlight various design pathways for system‐level integration of EAB sensors, spanning wearable, semi‐implantable to implantable and ingestible form factors. With integrated platforms now demonstrated, including in first‐in‐human studies, the focus is shifting from showing feasibility to addressing the challenges required for long‐term operation. In particular, improving stability, reducing biofouling, and enabling scalable multiplexing remain key priorities. Continued advances in aptamer design, antifouling materials, and low‐power electronics are expected to drive the development of integrated EAB sensors with prolonged functionality, enabling long‐term continuous molecular monitoring in vivo.

### 4.3 Bioresorbable In Vivo EAB Sensors

Advances in bioresorbable materials, particularly metals and polymers, have expanded their applications from traditional structural implants to functional electronic components [184, 185, 186]. These breakthroughs establish the material foundation for constructing bioresorbable sensors capable of reliable operation followed by safe bioresorption. By leveraging controlled bioresorption pathways, such systems can inherently eliminate the risks and costs associated with secondary retrieval surgeries [64, 187, 188]. Building upon these developments, recent studies have demonstrated the feasibility of bioresorbable electrochemical sensing platforms.

One early example was reported by Kim et al., who introduced a bioresorbable electrochemical in vivo biosensor composed of ultrathin silicon nanomembranes and iron catalyst nanoparticles [65]. This flexible sensor array enabled electrochemical detection of dopamine and demonstrated the conceptual feasibility of constructing in vivo biosensing systems entirely from bioresorbable materials (Figure 6a). The device operates by combining the semiconducting properties of silicon nanomembranes with the catalytic activity of iron nanoparticles to generate stable electrochemical signals. The entire system is bioresorbable, since silicon gradually hydrolyzes into metabolizable silicic acid species while iron undergoes oxidative dissolution into biocompatible ionic products. Accelerated degradation studies in PBS at pH 11 and 37°C revealed complete dissolution within 15 h, underscoring its bioresorption nature (Figure 6b). However, the study did not include in vivo bioresorption characterization and thus the translation of the observed bioresorption behavior into physiological conditions remains to be established. Despite this limitation, the work represents an important proof of concept for EAB sensors that combine functional operation with eventual bioresorption.

**FIGURE 6:**
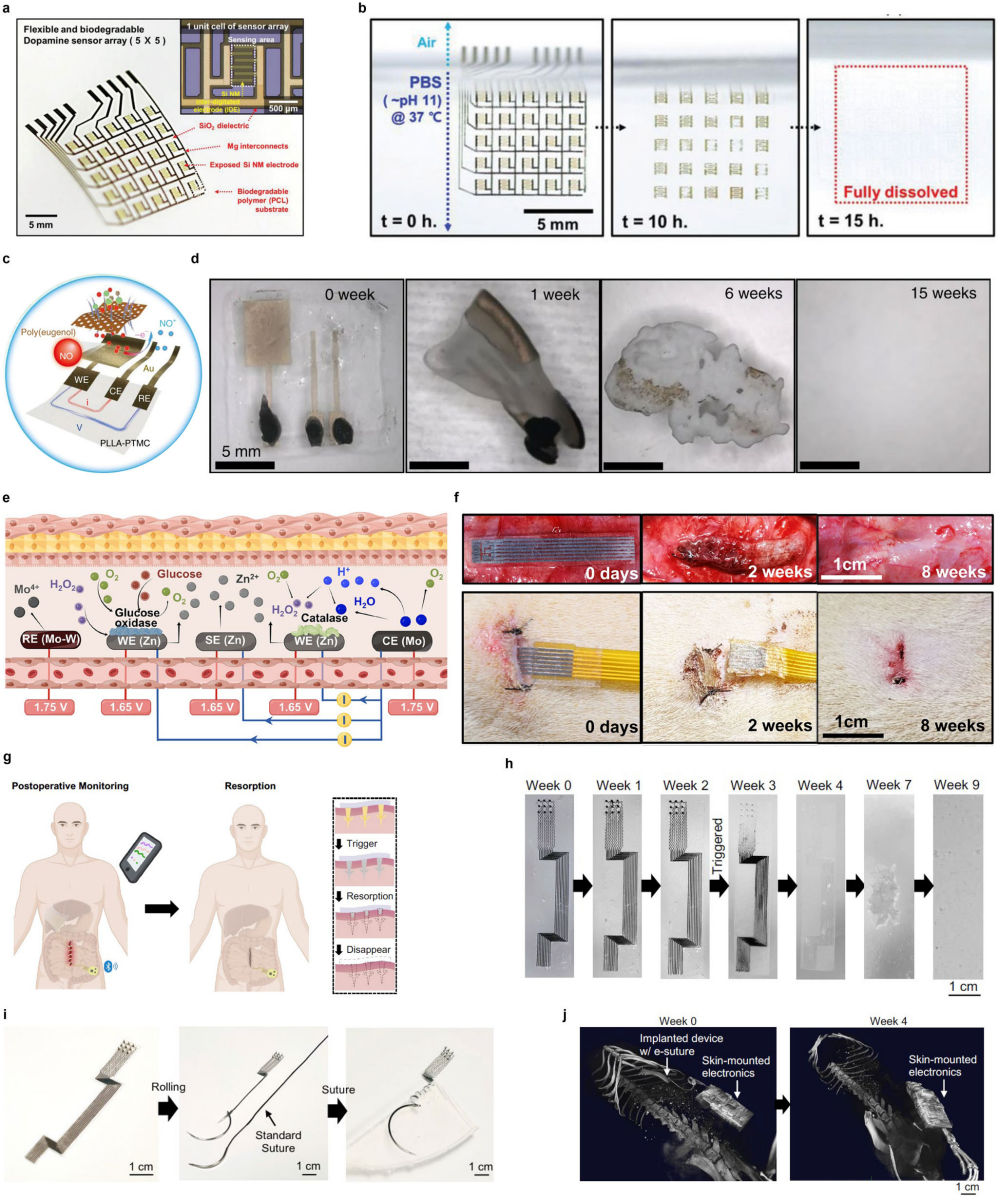
Bioresorbable in vivo sensors. (a,b) Bioresorbable electrochemical dopamine monitors. (a) Flexible dopamine‐sensing array fabricated using bioresorbable metals and polymers (Mo, Mg, Zn and PLGA/PLGA‐based substrates), enabling transient molecular monitoring without retrieval surgery. Reproduced with permission [65]. Copyright 2018, Wiley‐VCH GmbH. (b) Time‐lapse dissolution of the array under physiological pH (PBS, 37°C), showing progressive resorption of microelectrodes and complete resorption within 15 h. Reproduced with permission [65]. Copyright 2018, Wiley‐VCH GmbH. (c,d) Transient electrochemical nitric oxide sensor. (c) Polyglycolide/PLLA/PTMC‐based packaging ensures barrier function while permitting hydrolysis‐mediated device degradation. Reproduced under the terms of the CC‐BY license [66]. Copyright 2020, Springer Nature. (d) Degradation behavior tested in vivo over 15 weeks shows progressive loss of mechanical integrity and complete resorption of encapsulated devices. Adapted under the terms of the CC‐BY license [66]. Copyright 2020, Springer Nature. (e,f) Bioresorbable electrochemical devices for continuous glucose monitoring. (e) Transient electrochemical sensors integrate enzymatic glucose transduction with metal‐based electrodes composed of bioresorbable materials such as Zn, Mo and Mg for glucose and peroxide detection. Reproduced with permission [67]. Copyright 2023, American Association for the Advancement of Science. (f) In vivo studies in rats demonstrate progressive material resorption over 8 weeks. Adapted with permission [67]. Copyright 2023, American Association for the Advancement of Science. (g–j) Programmable bioresorbable electrochemical sensor for organ health monitoring. (g) A programmable bioresorbable in vivo sensor interfaces with an external electronics module to enable postoperative biochemical monitoring. Reproduced with permission [68]. Copyright 2026, Springer Nature. (h) In vitro characterization shows that the implanted module remains stable until triggered activation at Week 2, after which it undergoes progressive desorption over a 9‐week period. Reproduced with permission [68]. Copyright 2026, Springer Nature. (i) The electronic suture is fabricated by embedding molybdenum interconnects within PLGA filaments, producing a flexible construct that can be rolled, handled and deployed using standard surgical techniques. Reproduced with permission [68]. Copyright 2026, Springer Nature. (j) In vivo micro‐CT imaging confirms intact electrical interfacing between implanted bioresorbable sensors and skin‐mounted wireless electronics at Week 0, with visible suture resorption at Week 4. Reproduced with permission [68]. Copyright 2026, Springer Nature.

Li et al. developed a flexible and physically transient electrochemical sensor for real‐time nitric oxide monitoring [66]. The device was built on a copolymer substrate of poly(L‐lactic acid) and poly(trimethylene carbonate) (PLLA‐PTMC), integrated with ultrathin gold nanomembrane electrodes and a poly(eugenol) film to ensure electrochemical selectivity toward NO (Figure 6c). The bioresorption principle relies on the hydrolytic degradation of PLLA‐PTMC, combined with the progressive disintegration of the ultrathin Au and poly(eugenol) layers and eventual clearance of trace byproducts through metabolic pathways. Importantly, a biodegradable Mo‐based conductive paste was used as interconnects, which further supported complete system degradation. The bioresorption performance was systematically evaluated both in vitro and in vivo. Accelerated degradation studies in PBS at 65°C revealed progressive physical disappearance of the device, from intact structure at week 0, partial swelling and disintegration after 1 week, fragmentation into amorphous pieces by 6 weeks, and complete disappearance by 15 weeks (Figure 6d). Complementary in vivo implantation in rabbit joint cavities confirmed full resorption of the PLLA‐PTMC substrate and Au nanomembrane electrodes after 8 weeks, with histological analysis showing no residual materials or inflammatory reactions at the implantation site. These findings underscore that the bioresorption pathway is governed by substrate hydrolysis, nanomembrane disintegration and metabolic clearance, ensuring the safe bioresorption of the device following its functional lifetime.

More recently, Li et al. reported a fully printed and self‐compensated bioresorbable electrochemical device for continuous glucose monitoring [67]. The system was constructed from bioresorbable metallic pastes, including zinc working electrodes, molybdenum counter electrodes and molybdenum‐tungsten composite reference electrodes (Figure 6e). The principle of bioresorption derives from the inherent solubility of these metals in physiological environments. Zinc gradually oxidizes into Zn^2+^ ions, while molybdenum and tungsten undergo slow oxidative dissolution, together forming transient conductive pathways that ultimately disappear. A poly(lactic‐*co*‐glycolic acid) (PLGA) substrate and encapsulation further supported complete device resorption through hydrolytic degradation. The bioresorption performance was validated in both in vitro and in vivo studies. In PBS, the printed electrodes gradually disintegrated into small fragments within 2 months. When implanted subcutaneously in rats, the device exhibited progressive degradation and complete disappearance by 8 weeks (Figure 6f). Histological analyses, complete blood counts and blood chemistry tests confirmed that no residual material or inflammatory responses remained at the implantation site or in major organs. These findings establish a clear bioresorption trajectory driven by hydrolysis of the PLGA substrate, dissolution of Zn/Mo/W electrodes and metabolic clearance of ionic byproducts, underscoring the feasibility of constructing fully functional sensing systems that safely vanish following their operational lifetime.

Collectively, these studies highlight the promise of bioresorbable EAB sensors but also reveal persistent limitations. Current systems often rely on uncontrolled natural degradation, limiting tunability of device lifetimes and many still depend on non‐resorbable components such as potentiostat chips. Although chip‐free readout schemes such as ultrasound or inductor‐capacitor (LC) circuits have shown feasibility for specific parameters, they remain insufficient for electrochemical analysis, which rely on complicated interrogation waveforms [188, 189]. Moreover, most platforms remain confined to single‐analyte monitoring with limited in vivo validation, falling short of the integrated, programmable architectures envisioned for clinical translation.

To address these challenges, our group introduces a modular architecture in which a bioresorbable microneedle implant is connected to external electronics through an electronic suture (e‐suture), allowing stable operation followed by programmable bioresorption after clinical use (Figure 6g) [68]. Prior to triggering, the implanted microneedle array remains structurally intact for weeks. After applying a brief electrical trigger post‐implantation, the array commences degradation, ultimately achieving complete bioresorption, thereby validating the ability to externally command resorption kinetics (Figure 6h). The applied overpotential (>1.95 V) induces rapid corrosion of the metal coatings, exposing the underlying PLGA/Mo and accelerating hydrolytic resorption of the e‐suture and implant [190]. The electrical interconnects of the implant can be rolled into a bioresorbable e‐suture. The e‐suture functions as a surgical suture while providing electrical connection, eliminating the need for implanting non‐bioresorbable electronics that would otherwise require surgical retrieval (Figure 6i,j). By individually addressing each microneedle through the e‐suture, the system supports simultaneous biochemical and electrophysiological sensing across multiple sites, offering a clinically relevant pathway for scalable, multimodal monitoring of deep‐organ physiology.

## 5 Conclusion and Outlook

Despite substantial advances in sensor design, antifouling coatings, nanoengineered electrodes, surface chemistry, and system integration, in vivo EAB sensors still face five major challenges. 1) Extending operational lifetimes to clinically relevant timescales remains the central bottleneck. Although recent advances have enabled continuous monitoring over hours to days and, in select cases, up to 1 week, achieving stable operation over weeks to months is still required for real‐world clinical deployment. 2) Deciphering the dominant degradation mechanisms under in vivo conditions is essential to address this limitation at its root. A quantitative understanding of how electrochemical stress, monolayer instability, biofouling, and enzymatic degradation interact in vivo remains incomplete, limiting the ability to bridge in vitro characterization with in vivo performance. 3) Integrating complementary stabilization strategies into unified sensor designs is necessary to overcome the multifactorial nature of degradation. Existing approaches, including calibration‐based correction, XNA‐based probes, antifouling coatings, nanoengineered electrodes, and covalent immobilization, are often implemented in isolation, and their synergistic combination remains underexplored. 4) Developing fully integrated, clinically viable device systems is required for long‐term in vivo operation. This demands seamless integration of sensing interfaces with ultralow‐power electronics, wireless communication, power management, and biocompatible packaging. While recent progress in miniaturized wireless circuits has demonstrated the feasibility of fully integrated electrochemical systems, significant challenges remain. Bioresorbable devices represent an emerging class of systems that eliminate the need for surgical retrieval, yet most electronic components, particularly electrochemical readout circuits, are not fully bioresorbable. The e‐suture provides a practical interim solution by linking bioresorbable implants to external, non‐bioresorbable electronics, enabling functional operation without leaving permanent hardware behind [191, 192]. Continued advances in wireless communication strategies and bioresorbable semiconductor materials will be essential to ultimately realize fully bioresorbable continuous molecular monitoring systems. 5) Expanding EAB sensing to challenging targets remains an important frontier for broader applicability. Robust and reversible detection of proteins and other low‐abundance biomarkers is still difficult due to slow binding kinetics, limited signal gain, and increased susceptibility to fouling, thereby constraining the generalizability of the platform.

Ultimately, translating CMM from short‐term demonstrations to robust, long‐term clinical operation will require a seamless convergence of molecular engineering, materials design, device architecture, and wireless systems. Success in this domain would enable real‐time access to biochemical information that is currently inaccessible outside controlled laboratory settings, unlocking diagnostic and therapeutic opportunities that are not possible today. Future CMM platforms could transform perioperative care, guide precision dosing of narrow‐therapeutic‐index drugs, enable early detection of transplant rejection or organ injury, and provide continuous surveillance of inflammatory, metabolic, and neurological status. As these technologies mature, they hold the potential to fundamentally reshape clinical decision‐making by shifting from episodic laboratory tests to dynamic, personalized, and proactive management of human health.

## Author Contributions

S.L. and W.O. conceived the manuscript and wrote the draft. X.L. contributed to manuscript revision.

## Conflicts of Interest

The authors declare no competing interests.

## Data Availability

Data sharing not applicable to this article as no datasets were generated or analysed during the current study.
